# Linear or Cyclic? Theoretical Investigation of Astrophysically Relevant Magnesium‐Bearing MgC_*n*_H Carbon Chains and Related Isomers

**DOI:** 10.1002/jcc.70031

**Published:** 2025-01-13

**Authors:** A. Karolyna M. S. Gomes, Ricardo R. Oliveira, Thiago M. Cardozo, Felipe Fantuzzi

**Affiliations:** ^1^ Instituto de Química Universidade Federal do Rio de Janeiro Rio de Janeiro Brazil; ^2^ Insitut für Physik Ruhr‐Universität Bochum Bochum Germany; ^3^ Chemistry and Forensic Science, School of Natural Sciences University of Kent Canterbury UK

**Keywords:** astrochemistry, coupled cluster, DFT, magnesium, theoretical chemistry

## Abstract

Magnesium‐containing molecules, including MgC_2_H, MgC_4_H, and MgC_6_H, have been detected in the interstellar medium, largely facilitated by their high dipole moments. However, despite great efforts, MgC_2*m* + 1_H species remain elusive. Given the challenges in obtaining experimental data for these molecules, theoretical studies play a crucial role in guiding their detection. In this work, we present a theoretical analysis of MgC_*n*_H isomers (*n* = 4–7) using density functional theory and coupled‐cluster methods to identify low‐lying isomers and characterize their structural and electronic properties. Our findings reveal that across the entire series investigated, the global minimum geometry is linear for even values of *n*, whereas for odd values, a cyclic geometry is favored. Additionally, our calculations highlight the enhanced stability of anionic MgC_*n*_H^−^ systems, providing insights that could aid future astronomical detections in the interstellar medium.

## Graphical abstract

The global minimum energy structure of astrophysically relevant MgC_*n*_H systems is linear when *n* is even, and cyclic when *n* is odd.

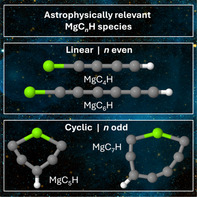

## 1 Introduction

To this day, more than 320 molecules have been identified in the interstellar medium (ISM) or circumstellar shells [1]. Given the presence of metals in these environments, the search for organometallic compounds has become increasingly important. Among the metals present in the ISM, magnesium stands out for its abundance and chemical significance. Notably, nearly half of the metal‐containing molecules detected around the star IRC +10216 contain magnesium [2, 3]. IRC +10216, a carbon‐rich circumstellar envelope, is renowned for its extraordinary molecular diversity and is one of the most chemically rich objects in the ISM. Magnesium is also the most prevalent metal in the solar system [4], which makes this element even more central in the quest for understanding our astrochemical origins.

Beyond its relevance in astrochemistry, which continues to fuel both theoretical and experimental exploration [5, 6], magnesium plays a vital role in biological systems on Earth. As an essential element for life, magnesium forms chelates with various biomolecules, including adenosine triphosphate (ATP), and activates enzymes involved in multiple stages of metabolic processes in living organisms [7, 8, 9, 10]. Magnesium is also a key component of chlorophyll, the molecule responsible for photosynthesis in plants [11]. In astrobiology, the detection of chlorophyll's absorption band at 670 nm is considered a potential biosignature, as its spectral signature could be preserved under Martian surface conditions, offering insights into the possibility of photosynthetic life beyond Earth [12].

Historically, the first magnesium‐bearing molecule discovered in the ISM was magnesium isocyanide (MgNC), detected in 1986 via microwave spectroscopy around IRC +10216 [13]. Following this discovery, magnesium‐containing compounds became prime targets for astronomical detection. Over time, several additional molecules were identified, including magnesium cyanide (MgCN) [14], HMgNC [15], MgC_2_H [16], MgC_3_N and MgC_4_H [17], MgC_5_N [18], MgC_6_H [18], and magnesium dicarbide (MgC_2_) [3]. Other recently detected Mg‐bearing molecules include HMgCCCN, MgC_4_H^+^, MgC_3_N^+^, MgC_6_H^+^, MgC_5_N^+^, and MgS [19, 20, 21].

For MgC_*n*_H‐type molecules, only those with even *n* values (*n* = 2, 4, 6) have been detected in IRC +10216, whereas analogs with an odd number of carbon atoms remain undetected. In a recent theoretical study, Panda et al. theoretically investigated isomers of MgC_3_H, and a total of 11 stationary points with doublet multiplicity (below 72 kcal/mol) were found [22]. Unlike the *n*‐even MgC_*n*_H species, for which both theoretical [23] and experimental [16, 17, 18] studies have shown linear global minima with terminal magnesium atoms, the global minimum of MgC_3_H exhibits a cyclic geometry [22]. This structural difference may explain why *n*‐odd species remain unobserved. Furthermore, the ISM contains species like C_3_H, C_3_H_3_^+^, Mg, and Mg^+^, which could potentially react to form cyclic MgC₃H compounds. This makes both c‐MgC₃H and c‐MgC₃H^+^ promising targets for future detection, particularly in IRC +10216. However, the detection of c‐MgC_3_H may be challenging due to its small dipole moment, which was calculated as 0.19 Debye [22].

Recently, various isomers of MgC_4_H were also theoretically predicted [24]. In addition to the previously known linear form with a terminal magnesium atom, new cyclic and linear structures were identified, all with energies less than 59 kcal/mol higher than the most stable form of MgC_4_H [24]. These findings suggest a trend in the global minimum geometries of MgC_*n*_H: for *n*‐even, the preferred structure is linear with a terminal magnesium atom, whereas for *n*‐odd, a cyclic structure is favored.

In this work, we provide a comprehensive analysis of the structure, stability, and bonding of MgC_*n*_H species, extending the study to systems with *n* up to 7. As part of a systematic investigation of astrophysically relevant molecules [25, 26, 27, 28, 29, 30], we identify low‐lying constitutional isomers for each MgC_*n*_H stoichiometry and compute their relative energies and thermodynamic stabilities using high‐accuracy computational methods, providing a detailed understanding of the structural preferences within the series. Finally, we propose new viable magnesium‐bearing candidates for future astrophysical detection.

## 2 Methods

Different isomeric starting structures of MgC_*n*_H (*n* = 4–7) were generated using the software CLUSTER 1.0 [31]. For all species, doublet multiplicities were assigned, and preliminary geometry optimizations were then performed at the UB3LYP/def2‐SVP level of theory [32, 33, 34, 35, 36]. Higher‐order multiplicities were also tested for selected isomers to ensure all possible low‐energy configurations were considered. From these calculations, low‐energy isomers—up to about 50 kcal/mol above the lowest energy structure—were selected for further refinement. These selected isomers were subsequently reoptimized at the UB3LYP/aug‐cc‐pVTZ level [37, 38, 39]. Vibrational frequency calculations at this level of theory confirmed that all reported geometries correspond to true minima on their respective potential energy surfaces (PESs). The use of DFT to determine minimum geometries was validated by its strong agreement with CCSD(T) structural parameters for MgC_4_H isomers, reported by Bâldea [23]. For increased accuracy in the electronic energies, single‐point calculations were performed at the optimized geometries using the CCSD(T) method, known for providing highly reliable energy values. These calculations employed the aug‐cc‐pV*X*Z basis sets, where *X* = T for *n* = 4, 5 and *X* = D for *n* = 6, 7. Quasi‐restricted orbitals (QROs) obtained from unrestricted Hartree–Fock (UHF) calculations were used to construct the reference determinants to minimize spin contamination effects. All dipole moments reported in this work were calculated at the CCSD(T) level.

The distinct isomers obtained in this work are labeled as ***n*x**, where ***n*** represents the number of carbon atoms in the MgC_*n*_H system. In turn, **x** is an alphabetical designation starting from **a**, with **a** denoting the lowest‐energy isomer. However, the linear carbon chain structures for MgC_5_H and MgC_7_H are labeled differently, as **5lin** and **7lin**, respectively. This distinction is made because these linear isomers exhibited exceptionally high spin contamination, with $\left\langle S^{2}\right\rangle$ values of 2.84 and 2.86, respectively. As a result, they were excluded from further analysis. Additional details are provided in the Supporting Information.

In addition to the structural optimizations, adiabatic and vertical ionization potentials (IPs) and electron affinities (EAs) were computed for selected species to gain further information on the electronic properties of MgC_*n*_H compounds. These properties were calculated as follows:

(1) $${\mathrm{IP}}_{\mathrm{ad}}=E^{+}\left(\text{cation}\right)-E^{0}\left(\text{neutral}\right)$$

(2) $${\mathrm{EA}}_{\mathrm{ad}}=E^{0}\left(\text{neutral}\right)-E^{-}\left(\text{anion}\right)$$

(3) $${\mathrm{IP}}_{\text{vert}}=E^{+}\left(\text{neutral}\right)-E^{0}\left(\text{neutral}\right)$$

(4) $${\mathrm{EA}}_{\text{vert}}=E^{0}\left(\text{neutral}\right)-E^{-}\left(\text{neutral}\right)$$

where $E^{q}\left(M\right)$ refers to the total energy of the system with charge q at the geometry M. The adiabatic ionization potential (${\mathrm{IP}}_{\mathrm{ad}}$) and electron affinity (${\mathrm{EA}}_{\mathrm{ad}}$) were determined by the energy difference between the cation or anion in their optimized geometries and the neutral molecule in its optimized geometry, with zero‐point energy (ZPE) corrections being considered. The vertical ionization potential (${\mathrm{IP}}_{\text{vert}}$) and electron affinity (${\mathrm{EA}}_{\text{vert}}$) were calculated by maintaining the geometry of the neutral molecule for the ionized species.

For selected cases, spin density calculations and bonding analyses were performed, with the former using Löwdin population analysis [40] and the latter employing the intrinsic bond orbital (IBO) method [41]. Additionally, selected fragmentation pathways involving the release of hydrogen and magnesium from neutral and anionic MgC_*n*_H structures were investigated. In total, 64 molecular structures with MgC_*n*_H stoichiometry were thoroughly investigated in this work, providing a detailed examination of the Mg‐bearing species. All electronic structure calculations were carried out using ORCA 5.0.3 [42, 43].

## 3 Results and Discussion

### 3.1 General Trends in the MgC_*n*_H Series

**FIGURE 1:**
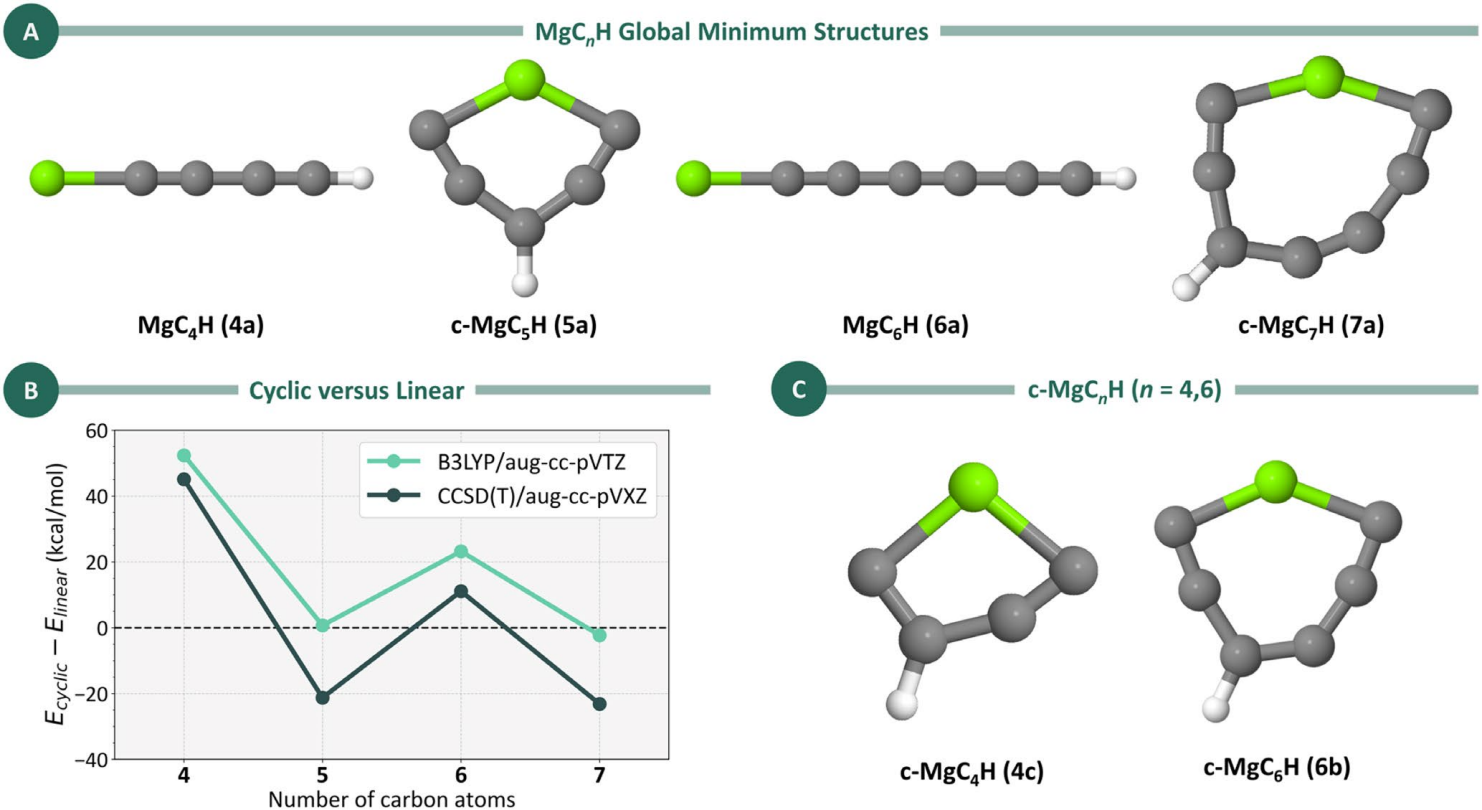
(A) Lowest‐energy isomers for MgC_*n*_H (*n* = 4–7) obtained at the CCSD(T)/aug‐cc‐pVXZ//UB3LYP/aug‐cc‐pVTZ level (*X* = T for *n* = 4 and 5 and *X* = D for *n* = 6 and 7). Isomers are labeled as described in the Section 2. (B) Energy differences (in kcal/mol) between cyclic and linear isomers for MgC_*n*_H (*n* = 4–7) calculated at UB3LYP/aug‐cc‐pVTZ (green) and CCSD(T)/aug‐cc‐pVXZ (teal). The cyclic isomers considered here are the most stable cyclic structures, where all non‐hydrogen atoms are incorporated into the ring. The linear MgC_5_H and MgC_7_H isomers considered here are labeled **5lin** and **7lin** (see text for details). (C) Most stable cyclic isomers for MgC_*n*_H (*n* = 4 and 6), where all non‐hydrogen atoms form part of the ring, obtained at the UB3LYP/aug‐cc‐pVXZ level.

Figure 1A shows the lowest energy structures obtained for MgC_*n*_H isomers with *n* = 4–7. These results reveal an alternating pattern in which cyclic structures are favored for odd‐numbered carbon chains, whereas linear structures are preferred for even‐numbered carbon chains. This trend is consistent with previously reported results for MgC_3_H, which also favors a cyclic geometry as the lowest energy structure [22].

This pattern becomes clearer when examining the energy differences between the most stable cyclic and linear isomers across the MgC_*n*_H series (*n* = 4–7), as shown in Figure 1B. For structures with an even number of carbons, the energy difference is positive, indicating a preference for linear geometries. Conversely, for the odd‐numbered systems, the energy difference is negative, favoring the cyclic structures c‐MgC_5_H (**5a**) and c‐MgC_7_H (**7a**) over the linear isomers **5lin** and **7lin**. The most stable cyclic isomers of MgC_4_H (**4a**) and MgC_6_H (**6a**), which were used in the plot of Figure 1B, are depicted in Figure 1C.

Figure 1B shows that DFT presents a bias toward the linear structures when compared to the CCSD(T) results. This is particularly dramatic in the case of MgC_5_H, where DFT incorrectly predicts the linear geometry as the lowest energy configuration. This tendency is larger for odd *n* because the linear geometry often results in significant spin contamination in the calculated spin states, leading to artificially lower energy estimates due to the increased variational freedom caused by this effect. In the following sections, we discuss each MgC_*n*_H series separately.

### 3.2 MgC_4_H

**FIGURE 2:**
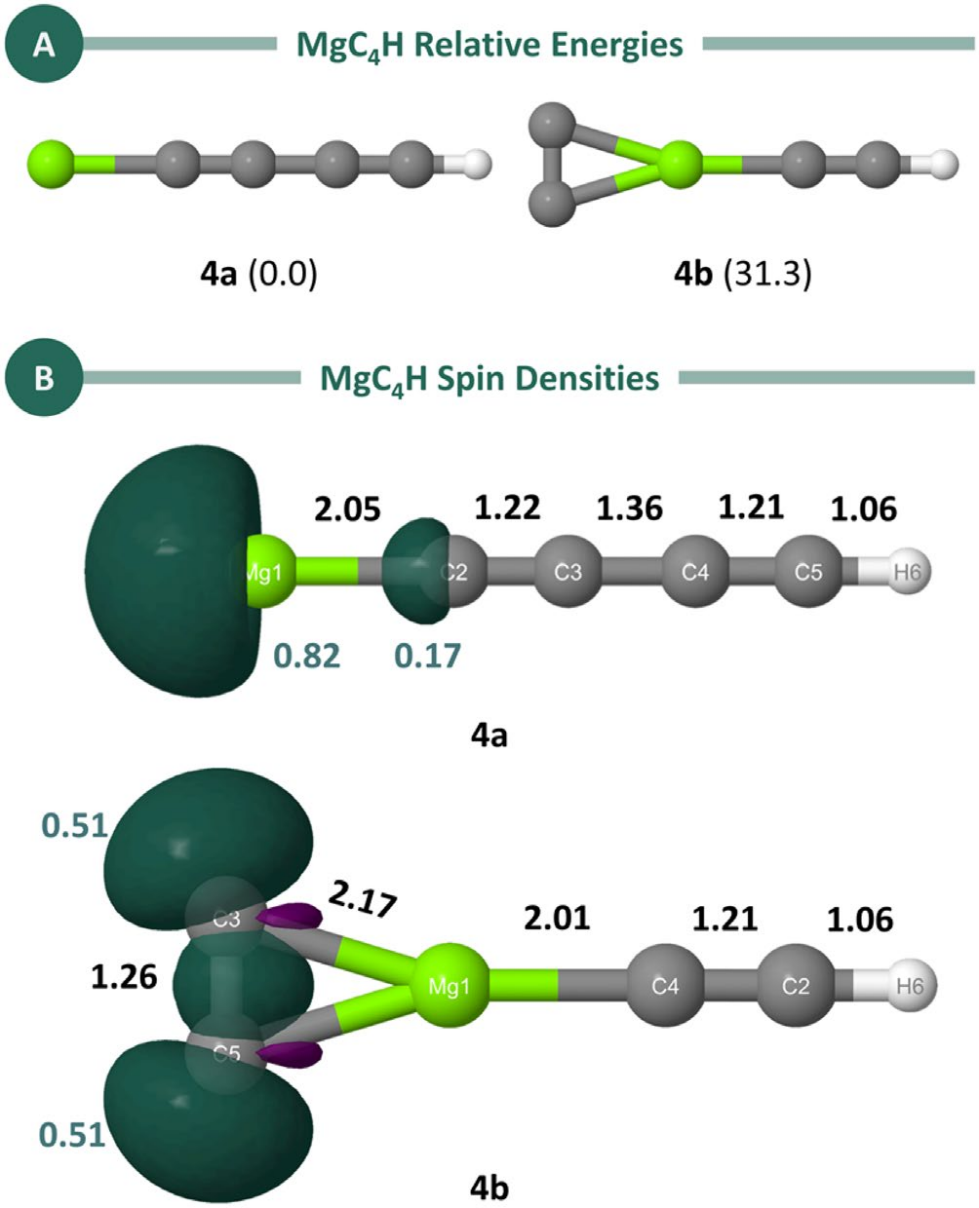
(A) Low‐energy isomers of MgC_4_H calculated at the UB3LYP/aug‐cc‐pVTZ level. Relative energies (in kcal/mol, shown in parentheses) were refined using the CCSD(T)/aug‐cc‐pVTZ method with ZPE corrections from the UB3LYP/aug‐cc‐pVTZ level. Dipole moments were also calculated at the CCSD(T)/aug‐cc‐pVTZ//UB3LYP/aug‐cc‐pVTZ level and are 2.2 D for **4a** and 0.8 D for **4b**. (B) Spin density plots for the low‐energy isomers of MgC_4_H. Bond lengths (in Å, black), were obtained at the UB3LYP/aug‐cc‐pVTZ level. The Löwdin population analysis of the spin density (teal) was calculated at the CCSD(T)/aug‐cc‐pVTZ level.

The lowest‐energy isomer identified for MgC₄H, referred to as Structure **4a**, is linear with an estimated dipole moment of approximately 2.2 Debye, as shown in Figure 2A. This isomer was previously studied by Bâldea [23], and its detection in IRC +10216 has already been reported by Cernicharo et al. [17]. The energies and structural parameters obtained in this work are in good agreement with those reported by Bâldea [23].

In Structure **4a**, it is possible to observe an alternation in carbon–carbon bond lengths, with the C2–C3 and C4–C5 bonds having lengths typical of triple bonds (1.20 Å), while the C3–C4 bond exhibits a length intermediate between typical double (1.34 Å) and single bonds (1.54 Å). The spin density plot (Figure 2B) reveals that the majority of the spin density is concentrated on the magnesium atom, a conclusion also reached by Bâldea [23] through natural bond orbital (NBO) [44] calculations.

The computational prediction of the second lowest energy isomer of MgC_4_H, Structure **4b**, was recently reported [24]. This isomer features a three‐membered C_2_Mg ring (Figure 2A) and lies Δ*E* = 31.6 kcal/mol above Structure **4a**. The spin density plot for this isomer indicates a notable difference from the linear isomer, with higher spin density concentrated along the carbon chain, particularly over the C3–C5 atoms, rather than on the magnesium atom. This suggests a transfer of electron density from the magnesium atom to the carbon chain.

In fact, the bond length observed for C3–C5 (1.2585 Å) falls between the values reported for neutral C_2_ [45] and anionic C_2_^−^ [46], but is closer to that of C_2_^−^. Furthermore, studies on MgC_2_ [3, 47, 48] also suggest an Mg^+^C_2_^−^ structure with a similar geometry to that observed in this work. According to these previous studies, the major interaction between the magnesium atom and the carbon chain is a Mg  → C_2_ charge transfer, with the cyclic MgC_2_ isomer stabilized relative to the linear congener due to a C_2_ → Mg backdonation, which can occur either in‐plane or out‐of‐plane [48].

It is worth noting that a cyclic MgC_4_H structure with C_2v_ symmetry was not identified, which is consistent with previous findings reported by Aoki at the DFT level [49]. Instead, c‐MgC_4_H distorts to a C_s_ structure, as shown in Figure 1C.

Regarding the charged analogs of these neutral species, the vertical and adiabatic IPs and EAs of the linear MgC_4_H Isomer **4a** have been previously reported by Bâldea [23]. Our results are in close agreement with those findings and confirm that, in all cases, the IPs and EAs are positive. These values are provided in Table S1.

The positive IP and EA values indicate a stability hierarchy, with the anionic MgC_4_H^−^ species being the most stable, followed by the neutral MgC_4_H structures, and the cationic MgC_4_H^+^ species as the least stable. Notably, the stabilization of the anionic form of **4b** is approximately twice as significant. Taken together, these results suggest that the still‐elusive MgC_4_H^−^ species, particularly the anion of **4b**, could be promising candidates for astronomical observation.

### 3.3 MgC_5_H

**FIGURE 3:**
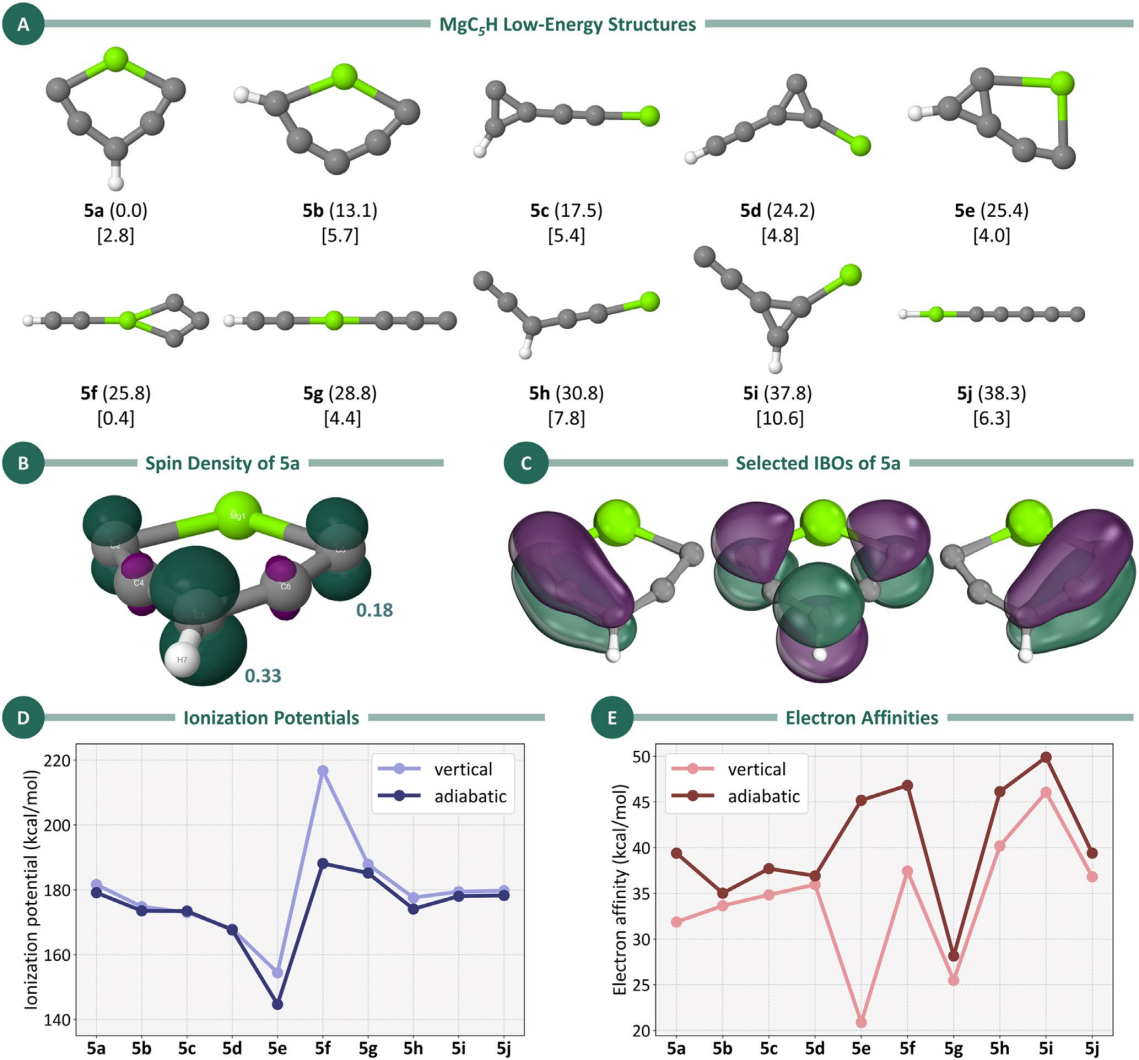
(A) Low‐energy isomers for MgC_5_H obtained at the UB3LYP/aug‐cc‐pVTZ level. Relative energies (in kcal/mol, in parentheses) were refined using the CCSD(T)/aug‐cc‐pVTZ method with ZPE corrections from the UB3LYP/aug‐cc‐pVTZ level. Dipole moments (in square brackets) were also determined at the CCSD(T)/aug‐cc‐pVTZ//UB3LYP/aug‐cc‐pVTZ level. (B) Spin density of the ground‐state geometry of MgC_5_H, with the Löwdin populational analysis of the spin density (CCSD(T)/aug‐cc‐pVTZ) shown in teal. (C) π‐Space IBOs for Structure **5a**. (D) Vertical and adiabatic ionization potentials (in kcal/mol) for MgC_5_H isomers. Geometries and ZPE were obtained at the UB3LYP/aug‐cc‐pVTZ level, with energies calculated at the UCCSD(T)/aug‐cc‐pVTZ level. (E) Vertical and adiabatic electron affinities (kcal/mol) for the isomers with the chemical formula MgC_5_H. Geometries and ZPE were obtained at the UB3LYP/aug‐cc‐pVTZ level, with energies calculated at the UCCSD(T)/aug‐cc‐pVTZ level.

For MgC_5_H, 10 distinct low‐energy isomers (plus **5lin**) were identified, and their geometries are shown in Figure 3A, along with ZPE–corrected energies and dipole moments. Our results indicate that, similar to MgC_3_H [22], the global minimum of MgC_5_H (**5a**) adopts a cyclic structure with C_2v_ symmetry, in contrast to the linear isomer observed for MgC_4_H [23]. Notably, many of the MgC₅H isomers exhibit calculated dipole moments exceeding 2 Debye. Given that species with smaller dipole moments—such as the CH radical, with a μ value of 1.46 Debye [50]—have been successfully detected by rotational spectroscopy [51], these findings suggest a promising potential for future experimental investigations of magnesium‐containing systems, particularly for targeted observations using radio telescopes.

Before diving into low‐lying isomers, we first focus on further characteristics of the global minimum **5a**. In terms of bond lengths (Figure S2), the Mg–C2 and Mg–C5 distances are consistent with typical Mg–C single bonds [52]. The C2–C4 and C5–C6 bonds display lengths intermediate between those of triple and double bonds, whereas the C3–C4 and C3–C6 bonds fall between double and single bond lengths. This suggests that multiple bonding schemes (i.e., resonance structures) might contribute to the final structure of this isomer. The spin density (Figure 3B) is concentrated among the *α* and *γ* carbon atoms, especially the latter.

Figure 3C shows selected IBOs for Structure **5a**. These orbitals reveal significant delocalization over three carbon atoms, indicating the presence of a delocalized π system across them. This finding is consistent with the structural parameters displayed in Figure 3B. Spin density plots for all structures depicted in Figure 3A were also generated and are available in Figure S4, highlighting the distribution of spin density across the various isomers, with some structures showing spin delocalization across the carbon chain, rather than concentrated on the magnesium atom. Although these higher‐energy isomers may be less abundant in astrochemical environments, determining their structures is crucial, as they could act as key intermediates in chemical reactions.

Out of the 10 lowest‐energy MgC_5_H isomers (excluding **5lin**), seven adopt cyclic configurations, with ring sizes varying from three to six atoms. Isomer **5b** (Δ*E* = 13.1 kcal/mol) stands out with its planar six‐membered ring, where the hydrogen atom is attached to a carbon in the *α* position relative to magnesium, unlike Isomer **5a**, in which the hydrogen occupies the *γ* position. This shift in hydrogen placement leads to a more compact ring structure, resulting in an increase in the C–Mg–C bond angle from 124.6^o^ in **5a** to 129.3^o^ in **5b**. Additionally, the distance between the magnesium atom and the *γ* carbon shortens from 2.82 Å in **5a** to 2.55 Å in **5b**.

Among the cyclic structures, four isomers—**5c** (Δ*E* = 17.5 kcal/mol), **5d** (Δ*E* = 24.2 kcal/mol), **5e** (Δ*E* = 25.4 kcal/mol), and **5i** (Δ*E* = 37.8 kcal/mol)—feature a distinctive three‐membered C_3_ ring. In Isomer **5c**, one of the carbon atoms in the ring is dicoordinate, with no attached substituents, whereas the other two carbon atoms are tricoordinate, with one bonded to a hydrogen atom and the other connected to a linear C_2_Mg fragment. Interestingly, in Isomer **5d**, the positions of the hydrogen and magnesium atoms are interchanged: the hydrogen atom migrates to the C_2_Mg fragment, whereas the magnesium atom occupies the former position of the hydrogen. This rearrangement results in a destabilization of 6.7 kcal/mol compared to **5c** and a modest decrease in dipole moment from 5.4 Debye in **5c** to 4.8 Debye in **5d**. It is worth highlighting that none of the low‐lying MgC_5_H isomers feature the C_5_^2−^ cluster with a planar tetracoordinate carbon, which was theoretically predicted for MgC₅ in earlier works [53, 54].

Isomer **5e** is unique among the 10 lowest‐energy structures, featuring a fused bicyclic arrangement. In this case, a three‐membered C_3_ ring is fused to a five‐membered C_5_Mg ring, with two carbon atoms participating at the junction of the two rings. This structure is planar and exhibits C_s_ symmetry. On the other hand, the least stable structure with a three‐membered ring is Isomer **5i**. In this isomer, all the carbon atoms in the C₃ ring are tricoordinate, bonded respectively to a hydrogen atom, a magnesium atom, and a C_2_ fragment. Among the 10 isomers, **5i** stands out with the highest dipole moment of 10.6 Debye.

Isomer **5f** (Δ*E* = 25.8 kcal/mol) is the only one with a four‐membered ring structure among the 10 lowest‐lying isomers. The four‐atom cycle adopts a rhombic geometry, reminiscent of the structure reported by Redondo et al. for MgC_3_ [55]. In this case, the remaining C₂H group is connected to the magnesium atom via one of the carbon atoms. Notably, its dipole moment is only 0.4 Debye, making it the least polar MgC_5_H isomer by a considerable margin.

The remaining isomers—**5lin** (Δ*E* = 21.3 kcal/mol), **5g** (Δ*E* = 28.8 kcal/mol), **5h** (Δ*E* = 30.8 kcal/mol), and **5j** (Δ*E* = 38.3 kcal/mol)—are open‐chain structures, with **5g** and **5j** adopting linear configurations. Isomer **5g** is characterized by a linear C_3_MgC_2_H arrangement, whereas **5j** consists of a C_5_MgH chain. Other potential linear structures were found to be too high in energy and were therefore excluded from further analysis. In contrast, Isomer **5h** features an open‐chain MgC_5_ structure, where the central carbon of the C_5_ chain is hydrogenated.

Optimized geometries for the cations and anions associated with the distinct MgC_5_H isomers are provided in Figure S5. These geometries were computed at the UB3LYP/aug‐cc‐pVTZ level of theory. The figure highlights the most significant variations in bond lengths and angles observed during the transitions between neutral, cationic, and anionic states. Particularly, bonds and angles involving the magnesium atom undergo the most significant changes. In the case of the lowest‐energy isomer, changes in C–C bond lengths are also observed, which aligns with the spin density distribution predominantly located along the carbon chain.

The adiabatic and vertical IPs and EAs for all structures shown in Figure 3A are presented in Figure 3D,E, respectively, with the numerical values provided in Table S2. The adiabatic IP values range from 140 to 190 kcal/mol, with **5f** showing the highest value and **5e** the lowest. Interestingly, these two isomers also exhibit the largest deviations between their vertical and adiabatic IP values, whereas the other isomers have vertical IPs closely matching their adiabatic counterparts. This indicates that, except for **5e** and **5f**, the geometries of the neutral MgC_5_H isomers are quite similar to their corresponding cationic forms.

In terms of EAs, adiabatic EA values range from 27 to 50 kcal/mol, with **5g** showing the lowest EA and **5i** the highest. Once more, **5e** stands out by displaying the largest difference between its vertical and adiabatic EA values, similar to the case of the IP. This suggests that both the cationic and anionic forms of **5e** undergo significant geometric changes compared to their neutral structure.

Finally, it is also worth mentioning that, as observed for MgC_4_H, the IP and EA values for all MgC_5_H cases are positive. Again, this indicates that the anionic species are the most stable, followed by the neutral structures, with the cationic species being the least stable. These findings suggest the potential viability of detecting MgC_5_H^−^ species. We hope these results will inspire further efforts to detect anionic magnesium‐bearing carbon chains in the ISM, particularly in IRC +10216, where such species remain elusive.

### 3.4 MgC_6_H

**FIGURE 4:**
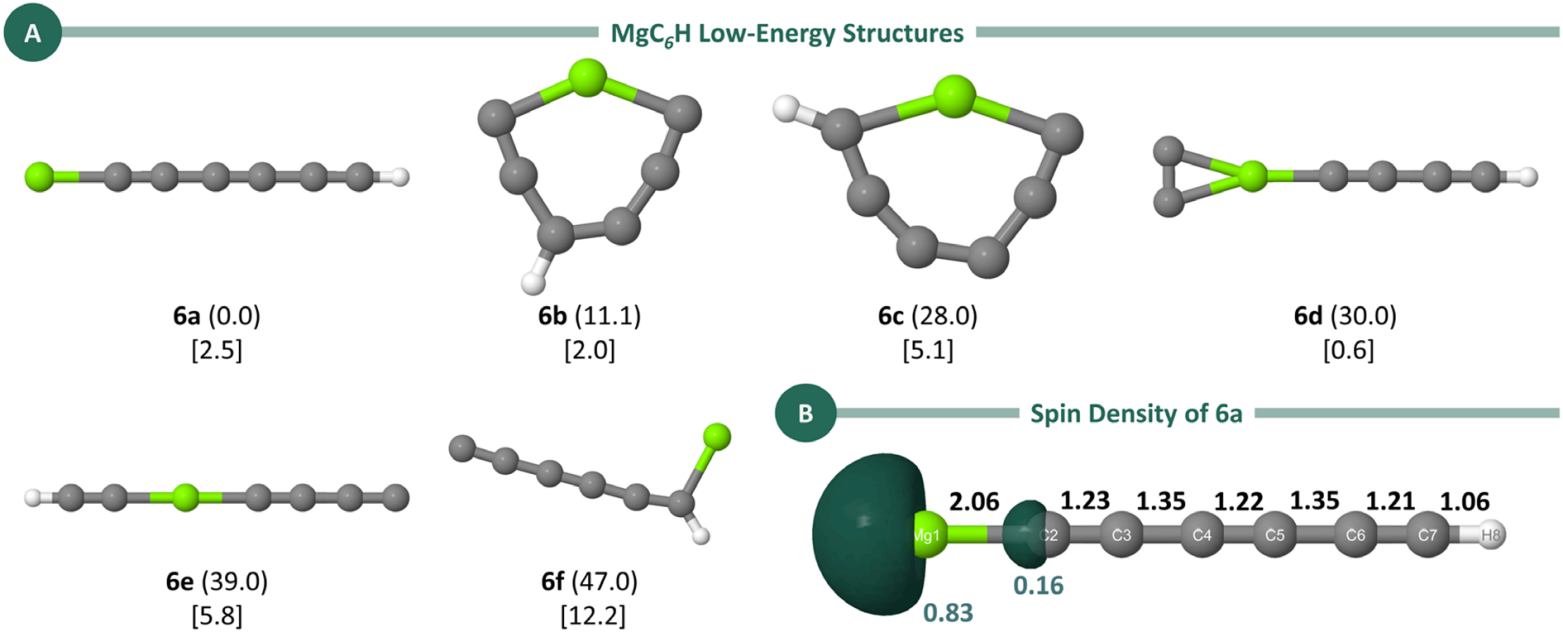
(A) Low‐energy isomers for MgC_6_H obtained at the UB3LYP/aug‐cc‐pVTZ level. Relative energies (in kcal/mol, in parentheses) were refined using the CCSD(T)/aug‐cc‐pVTZ method with ZPE corrections from the UB3LYP/aug‐cc‐pVTZ level. Dipole moments (in square brackets) were also obtained at the CCSD(T)/aug‐cc‐pVTZ//UB3LYP/aug‐cc‐pVTZ level. (B) Spin density distribution for Isomer **6a** at the CCSD(T) level. Bond lengths (in Å) are given in black, while the Löwdin population analysis of the spin density, calculated at the CCSD(T)/aug‐cc‐pVTZ level, is shown in teal.

For MgC_6_H, six distinct structures were identified, as shown in Figure 4A. Consistent with previously reported results for MgC_4_H, the global minimum for MgC_6_H features a linear structure with a terminal magnesium atom.

We begin by discussing the structural and bonding characteristics of Isomer **6a**. Upon examining the geometric parameters of this linear structure (Figure 4B), a similar alternation of bond lengths to that observed in MgC_4_H is evident. Specifically, the C2–C5, C7–C3, and C4–C6 bonds exhibit lengths typical of triple bonds (~1.20 Å), whereas the C2–C7 and C3–C6 bonds have longer bond lengths. However, these lengths are shorter than those observed in MgC_4_H, suggesting double bond characteristics (~1.34 Å) rather than single bonds (~1.54 Å).

The spin density plot and its population analysis for the linear structure are also presented in Figure 4B. These results show that, similar to MgC_4_H, the majority of the spin density is localized on the magnesium atom. Spin density plots for the other low‐lying isomers of MgC_6_H are provided in Figure S6.

In contrast to the linear structure of **6a**, the next three low‐lying isomers adopt cyclic configurations. Isomer **6b** (Δ*E* = 11.1 kcal/mol) features a seven‐membered MgC_6_ ring with C_s_ symmetry, where the hydrogen atom is bonded to one of the carbons in the *para* position relative to magnesium. Similarly, isomer **6c** (Δ*E* = 28.0 kcal/mol) also contains a seven‐membered ring, but with the hydrogen atom attached to a carbon in the *ortho* position to magnesium. No low‐lying structures with six‐, five‐, or four‐membered rings were identified. The final cyclic structure, Isomer **6d** (Δ*E* = 30.0 kcal/mol), consists of a three‐membered MgC_2_ ring, with a C_4_H group bonded to the magnesium atom. Among the two low‐lying open‐chain structures, Isomer **6e** (Δ*E* = 39.0 kcal/mol) features a linear C_4_MgC_2_H chain, whereas **Isomer 6f** (Δ*E* = 47.0 kcal/mol) has a quasi‐linear C_6_ unit, where one of the terminal carbon atoms is tricoordinate and bonded to both the hydrogen and magnesium atoms. It is worth noting that, except for Isomer **6d** (0.6 Debye), all MgC_6_H isomers exhibit dipole moments of 2.0 Debye or higher, with Isomer **6f** having the largest dipole moment at 12.2 Debye.

### 3.5 MgC_7_H

**FIGURE 5:**
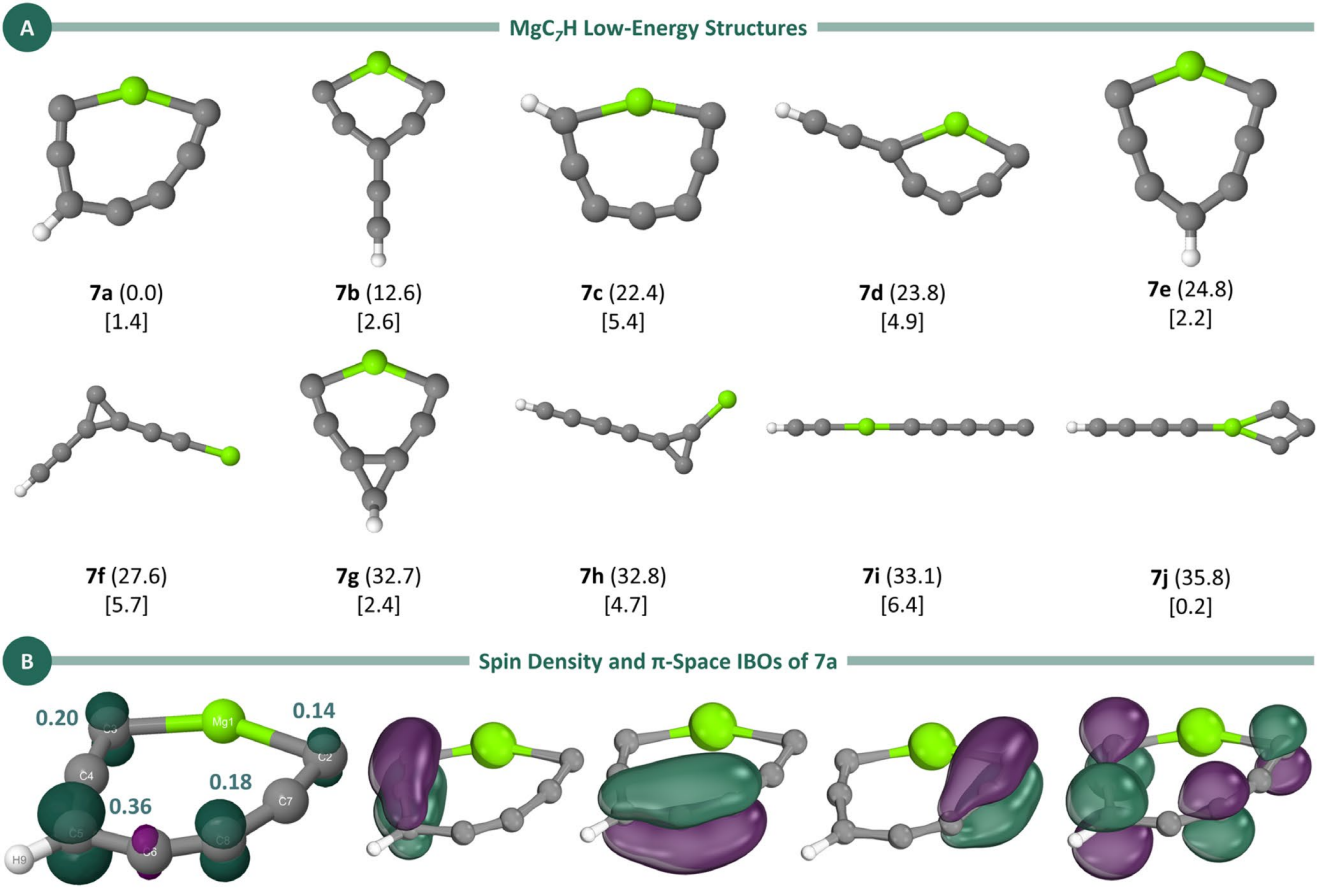
(A) Low‐energy isomers for MgC_7_H calculated at the UB3LYP/aug‐cc‐pVTZ level. Relative energies (in kcal/mol, in parentheses) were refined using the CCSD(T)/aug‐cc‐pVTZ method with ZPE corrections from the UB3LYP/aug‐cc‐pVTZ level. Dipole moments (in square brackets) were also obtained at the CCSD(T)/aug‐cc‐pVTZ//UB3LYP/aug‐cc‐pVTZ level. (B) Spin density and π‐space IBOs for Structure **7a**. The Löwdin population analysis of the spin density, calculated at the CCSD(T) level, is shown in teal.

The final stoichiometry examined in this study is MgC₇H, for which we identified 18 isomers within a 50 kcal/mol energy threshold. The first 10 of these isomers are presented in Figure 5A, whereas the others are shown in the Supporting Information. Consistent with the findings for MgC_5_H and MgC_3_H [22], the lowest‐energy structure for MgC_7_H (**7a**) is also cyclic. However, unlike the previous cases, this cyclic structure exhibits C_s_ symmetry rather than C_2v_, with the hydrogen atom attached to a carbon atom in the *γ* position relative to magnesium. This isomer appears to be analogous to the one found for MgC_5_H, with the addition of an ethylene group to one side of the carbon chain. This similarity is supported by comparable bond lengths and spin density patterns.

Figure 5B displays the geometric parameters, spin density plot, and Löwdin population analysis for the spin density. As with MgC_5_H, the lowest‐energy isomer of MgC_7_H exhibits an alternation of C–C bond lengths along the carbon chain. These bond lengths fall between those typical of single and double bonds or double and triple bonds. Additionally, the spin density shows a nodal plane, with the highest concentration located on carbon atom C5 and significant contributions on atoms C2, C3, and C8.

In addition to **7a**, the Isomers **7b** (Δ*E* = 12.6 kcal/mol), **7c** (Δ*E* = 22.4 kcal/mol), **7d** (Δ*E* = 23.8 kcal/mol), **7e** (Δ*E* = 24.8 kcal/mol), **7f** (Δ*E* = 27.6 kcal/mol), **7g** (Δ*E* = 32.7 kcal/mol), **7h** (Δ*E* = 32.8 kcal/mol), and **7j** (Δ*E* = 35.8 kcal/mol) are also cyclic. Three of these Isomers—**7a**, **7c** and **7e**—feature eight‐membered rings in planar structures, with **7c** having the hydrogen attached to a carbon atom *ortho* to magnesium, and **7e** displaying a 1,5‐substitution pattern. In contrast, **7b** and **7d** each feature a six‐membered ring with a C_2_H substituent at one of the carbon atoms—*γ* to magnesium in **7b** and *α* to magnesium in **7d**.

The Isomers **7f**, **7g** and **7h** possess a three‐membered C_3_ ring, whereas **7j** features a four‐membered C_3_Mg ring reminiscent of **5f**. In turn, **7g** consists of a bicyclic structure where a seven‐membered MgC_6_ ring is fused to the C_3_ ring via two carbon atoms, with the hydrogen atom connected to the third carbon of the C_3_ cycle.

Finally, the two lowest‐energy open‐chain structures are **7lin** (Δ*E* = 23.2 kcal/mol) and **7i** (Δ*E* = 33.1 kcal/mol), the latter containing a linear C_5_MgC_2_H chain. Among these structures, the dipole moments range from 0.2 Debye for **7j** to 6.4 Debye for **7i**.

### 3.6 Fragmentation Studies

A critical factor influencing the likelihood of detecting these molecules is their stability against fragmentation. To address this, we performed a systematic fragmentation analysis of the ***n*a** isomers (*n* = 4–7), including their anionic counterparts. Although fragmentation can occur through various pathways, this study focuses on the loss of hydrogen and magnesium for the neutral species and magnesium loss for the anionic forms. These pathways, identified as the lowest‐energy dissociation reactions for the linear MgC_4_H in previous studies [23], are particularly relevant to our analysis.

As shown in Table S3, all dissociation enthalpies are positive, ranging from +23.0 kcal/mol (**6a** ^**−**^ → C_6_H^−^ + Mg^0^) to +145.8 kcal/mol (**6a** → MgC_6_ + H). For the linear Isomers **4a** and **6a**, the Mg–C bond is more prone to breaking compared to the C–H bond. However, this trend does not apply to the cyclic species, where the C–H bond is more susceptible to fragmentation than the Mg–C bond. This behavior arises because, in cyclic species, the magnesium atom interacts with two carbon atoms, making magnesium dissociation less favorable.

For the anionic species, a similar pattern is observed: the cyclic structures exhibit greater stability than their linear counterparts with respect to magnesium loss. This enhanced stability suggests that cyclic anionic species could be particularly promising candidates for experimental observation.

### 3.7 MgC_*n*_H Versus MgC_*n*_ ^−^

When examining the full dataset of MgC_*n*_H species investigated in this work, along with data reported by Panda et al. [22] for MgC_3_H, a clear trend emerges: for even *n*, the linear structure is energetically favored, whereas for odd *n*, the cyclic structure is preferred. Interestingly, for even *n*, the most stable structure consistently appears to be linear with the spin density centered on the magnesium atom. This finding contrasts with prior studies on isoelectronic species of the MgC_n_^−^ type, where cyclic structures were reported to be more stable for even *n*, and linear structures for odd *n* [55]. The discrepancy likely arises from differences in the computational methods employed. More recent high‐level CC calculations for MgC_4_H and MgC_3_H align with the trends observed in this work, further supporting the validity of the current results [22, 23].

## 4 Conclusions

In this study, we investigated the low‐energy isomeric structures of MgC_*n*_H (*n* = 4–7) to explore the significance of magnesium in interstellar chemistry and the absence of MgC_*n*_H compounds with odd carbon numbers in the interstellar medium. The results reveal distinct structural characteristics depending on whether *n* is even or odd. For even *n*, the lowest‐energy isomers, including two and six low‐energy isomers for *n* = 4 and 6, respectively, were linear MgC_*n*_H chains exhibiting alternating C–C bond lengths akin to polyynic structures, though with shorter bond lengths indicating a partial cumulenic character. In contrast, odd *n* isomers displayed greater structural diversity, with 10 and 17 low‐energy isomers found for MgC_5_H and MgC_7_H, surpassing the number of isomers found for their even *n* counterparts. This diversity arises from the stability of linear structures in even *n* cases, where an unpaired electron on the magnesium atom is feasible, whereas for odd *n*, cyclic structures are favored. The lowest‐energy MgC_5_H structure adopts a cyclic geometry with C_2v_ symmetry, similar to that of MgC_3_H. In contrast, MgC_7_H exhibits a comparable cyclic form, but without C_2v_ symmetry, as the hydrogen atom is preferentially positioned *γ* to the magnesium atom, facilitating the formation of three C ≡ C triple bonds. The IBO analysis for MgC_7_H reveals a bonding pattern closely resembling that observed in the MgC_5_H species. In both cases, the IBOs display significant electron delocalization along the carbon backbone. Additionally, all MgC_5_H isomers demonstrated positive EA, suggesting the formation of stable anions under astrochemical conditions. Many isomers exhibited dipole moments suitable for rotational spectroscopy detection, indicating a promising avenue for further experimental studies and radio telescope observations.

## Supporting information


**Data S1.** Supporting Information.

## Data Availability

The data that supports the findings of this study are available in the supplementary material of this article.
