# Supplementary material for: Linear or Cyclic? Theoretical Investigation of Astrophysically Relevant Magnesium‐Bearing MgC n H Carbon Chains and Related Isomers
Source: J Comput Chem. 2025 Jan 13;46(2):e70031. doi: 10.1002/jcc.70031 (PMC11727008; doi:10.1002/jcc.70031)
Supplement: Supplementary file 1 — Data S1. Supporting Information. [file JCC-46-0-s001.docx]

Supporting Information for

**Linear or cyclic? Theoretical investigation of astrophysically relevant magnesium-bearing MgC*_n_*H carbon chains and related isomers**

**A. Karolyna M. S. Gomes,^1,2^ Ricardo R. Oliveira,^1^ Thiago M. Cardozo,^1^ Felipe Fantuzzi^3^**

Correspondence to: Thiago M. Cardozo ([thiago@iq.ufrj.br](mailto:thiago@iq.ufrj.br)); Felipe Fantuzzi (E-mail: [f.fantuzzi@kent.ac.uk](mailto:f.fantuzzi@kent.ac.uk))

^1^ Instituto de Química, Universidade Federal do Rio de Janeiro, Av. Athos da Silveira Ramos 149, Rio de Janeiro 21941-909, Brazil.

^2^ Insitut für Physik, Ruhr-Universität Bochum, Universitätstraße 150, Bochum 44801, Germany.

^3^ Chemistry and Forensic Science, School of Natural Sciences, University of Kent, Park Wood Rd, Canterbury CT2 7NH, UK.

**Additional computational data**

**Table S1** Adiabatic and vertical IP and EA (kcal/mol) for isomers with the chemical formula MgC_4_H. Geometries and ZPE obtained at the UB3LYP/aug-cc-pVTZ level and energy calculated at the CCSD(T)/aug-cc-pVTZ level. Values in (*) were obtained by Bâldea 2020 (*Mon. Not. R. Astron. Soc.* **2020**, *498*, 4316–4326).

|  | $\boldsymbol{IP}_{\boldsymbol{adiab}}$ | $\boldsymbol{IP}_{\boldsymbol{vertical}}$ | $\boldsymbol{EA}_{\boldsymbol{adiab}}$ | $\boldsymbol{EA}_{\boldsymbol{vertical}}$ |
| --- | --- | --- | --- | --- |
| **4a** | 168.1 | 168.3 | 31.8 | 29.3 |
| **4a^(*)^** | 166.1 | 172.0 | 34.6 | 30.8 |
| **4b** | 250.6 | 247.7 | 69.3 | 66.3 |


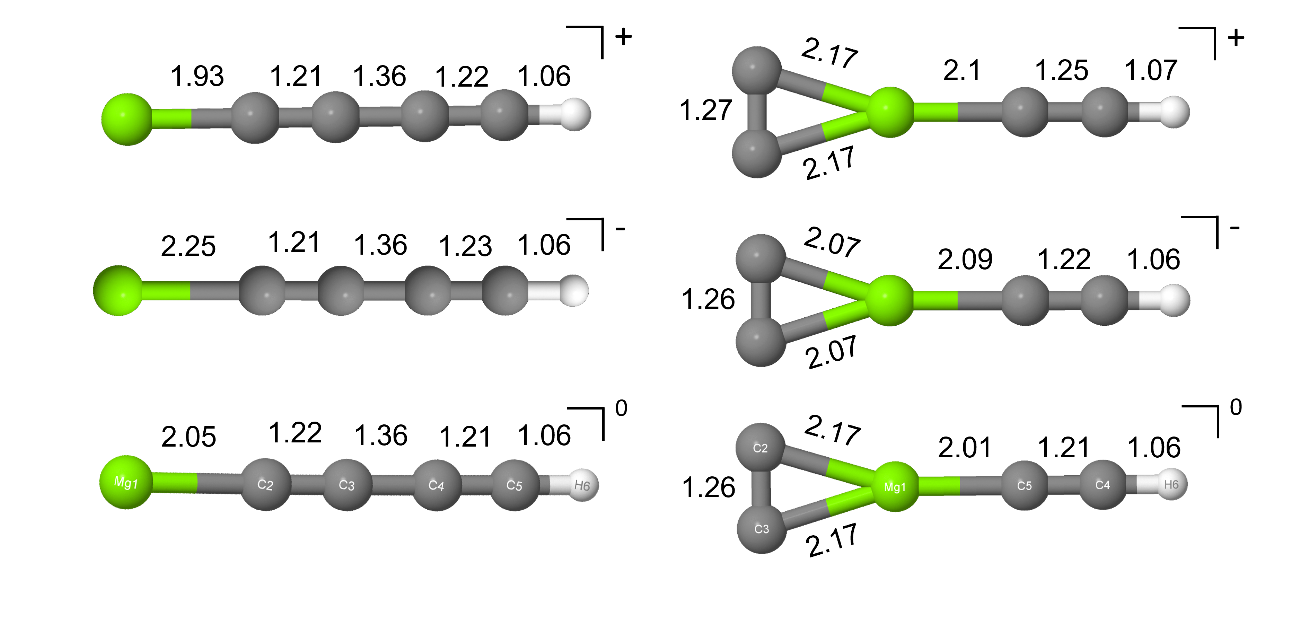


**Figure S1** Cations and anions obtained for the chemical formula MgC_4_H. Geometry optimizations were performed at the UB3LYP/aug-cc-pVTZ level. Bond distances in angstroms.


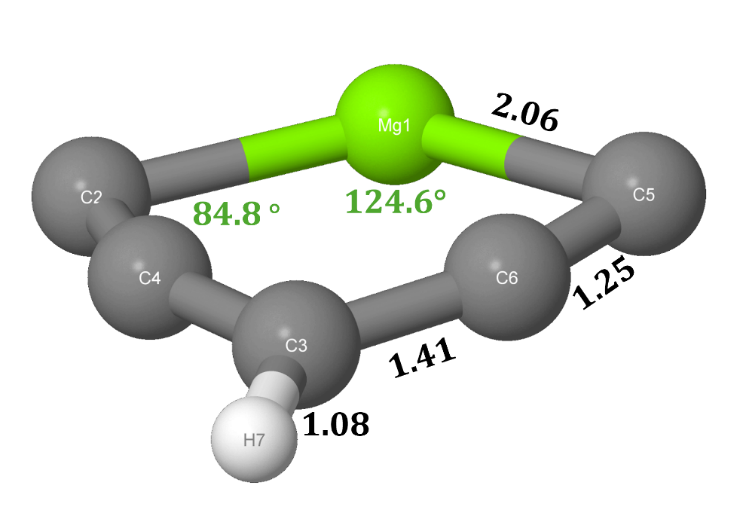


Figure S2 Structural parameters obtained at the UB3LYP/aug-cc-pVTZ level for structure 5a. Bond lengths (Å) are reported in black, bond angles (degrees) in green.


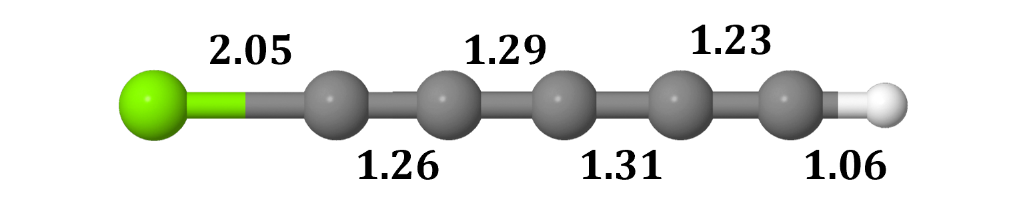


Figure S3 Structural parameters obtained at the UB3LYP/aug-cc-pVTZ level for structure 5lin. Bond lengths (Å) are reported in black.


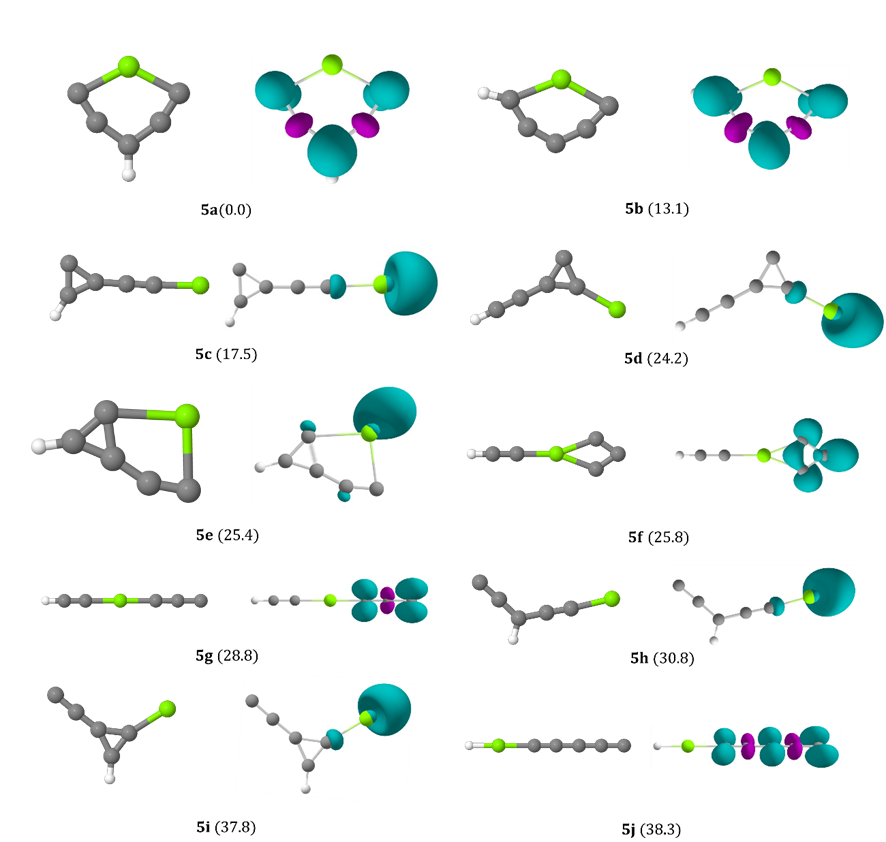


Figure S4 Spin density at the CCSD(T)/aug-cc-pVTZ//UB3LYP/aug-cc-pVTZ level for the low-energy isomers with the chemical formula MgC_5_H.


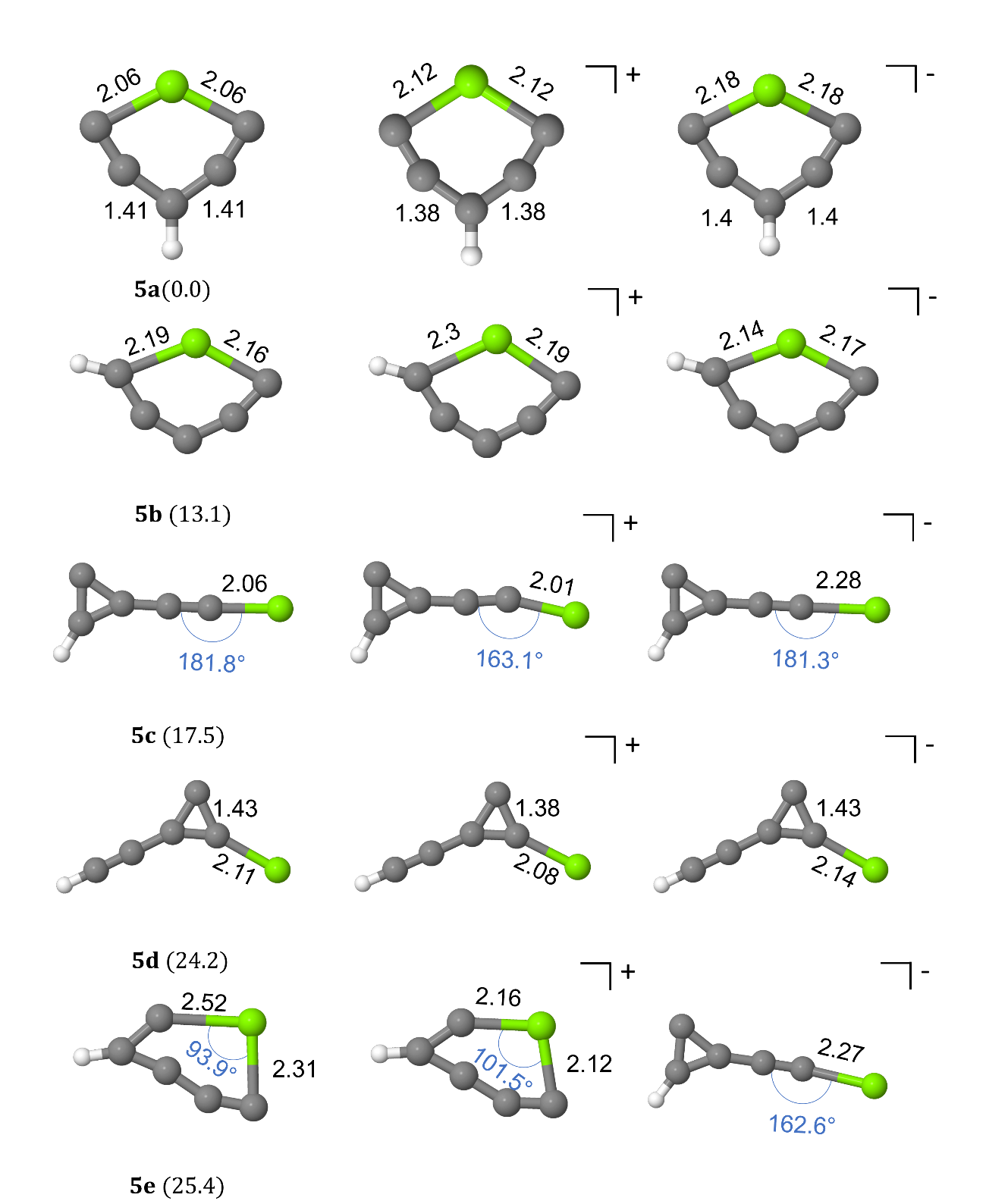


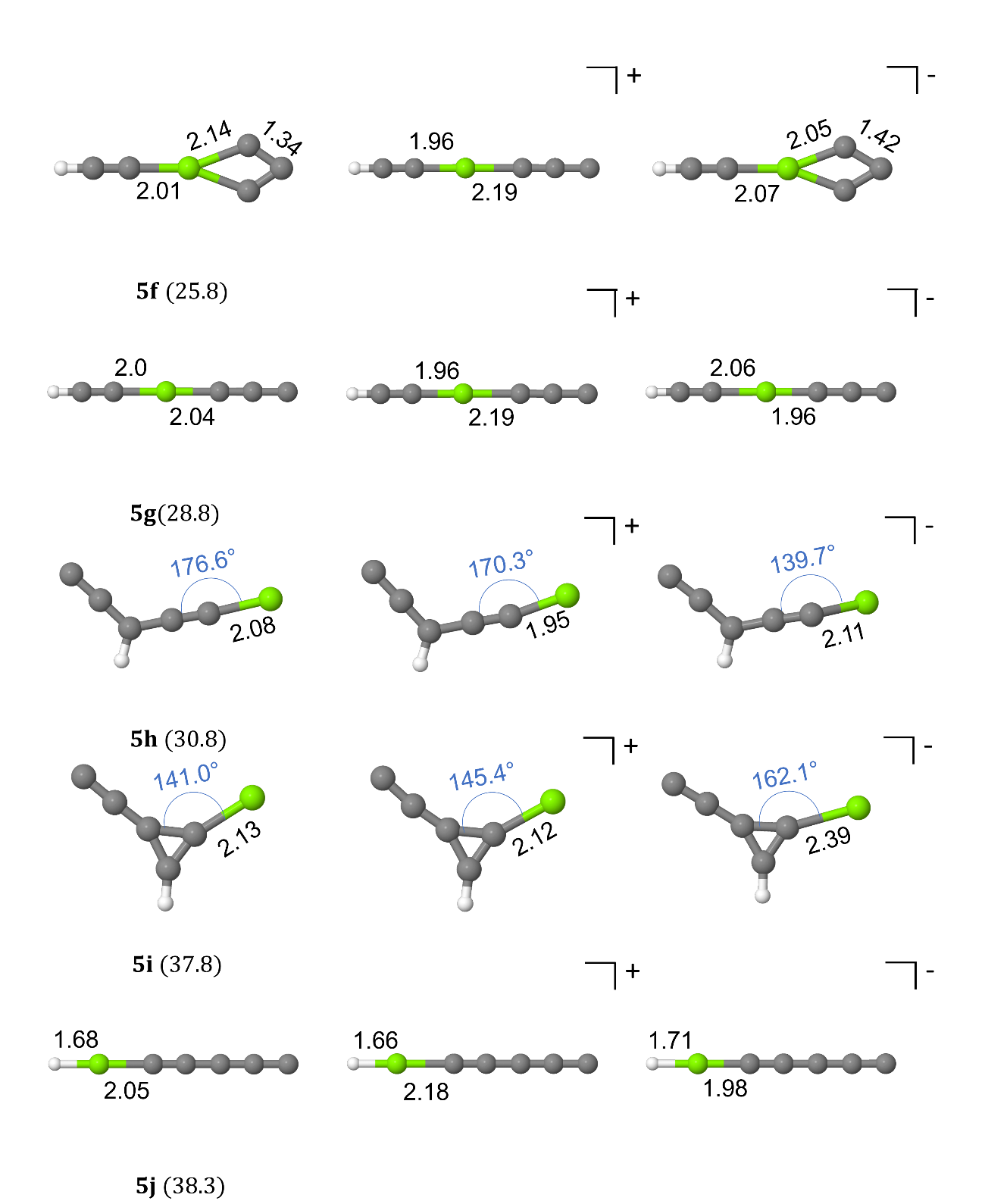


**Figure S5** Cations and anions obtained for the chemical formula MgC_5_H. Geometry optimizations were performed at the UB3LYP/aug-cc-pVTZ level. Bond distances in angstroms and angles in degrees.

**Table S2** Adiabatic and vertical IP and EA (kcal/mol) for the isomers with the chemical formula MgC_5_H. Geometries and ZPE obtained at the UB3LYP/aug-cc-pVTZ level and energy calculated at the CCSD(T)/aug-cc-pVTZ level.

|  | $\boldsymbol{IP}_{\boldsymbol{adiab}}$ | $\boldsymbol{IP}_{\boldsymbol{vertical}}$ | $\boldsymbol{EA}_{\boldsymbol{adiab}}$ | $\boldsymbol{EA}_{\boldsymbol{vertical}}$ |
| --- | --- | --- | --- | --- |
| **5a** | 179.11 | 181.63 | 39.39 | 31.86 |
| **5b** | 173.51 | 174.80 | 35.01 | 33.64 |
| **5c** | 173.43 | 173.03 | 37.70 | 34.83 |
| **5d** | 167.63 | 167.89 | 36.91 | 35.95 |
| **5e** | 144.66 | 154.42 | 45.17 | 20.87 |
| **5f** | 188.13 | 216.78 | 46.81 | 37.43 |
| **5g** | 185.16 | 187.84 | 28.15 | 25.47 |
| **5h** | 174.07 | 177.56 | 46.12 | 40.18 |
| **5i** | 178.03 | 179.42 | 49.89 | 46.05 |
| **5j** | 178.26 | 179.74 | 39.40 | 36.81 |


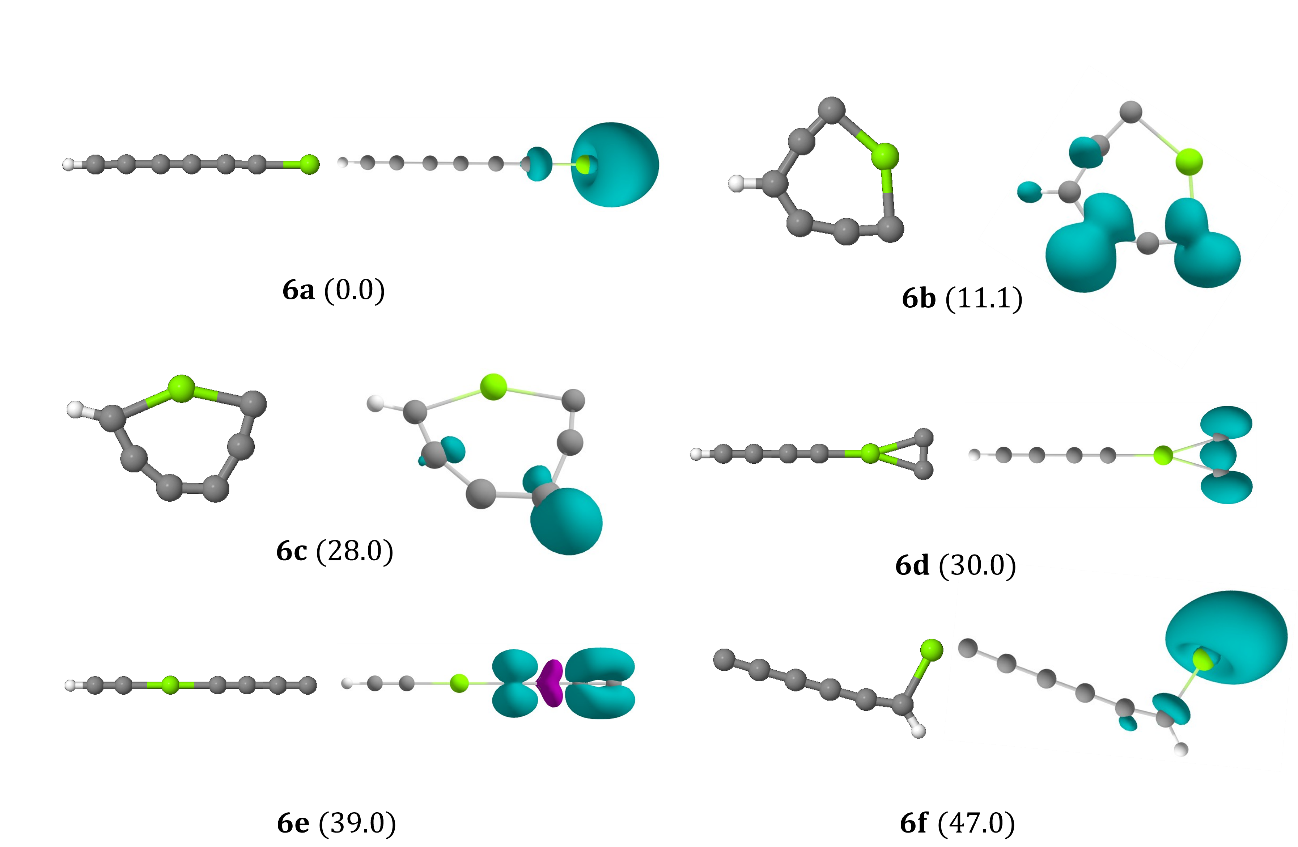


Figure S6 Spin density at the CCSD(T)/aug-cc-pVTZ//UB3LYP/aug-cc-pVTZ level of theory for the low-energy isomers with the chemical formula MgC_6_H.


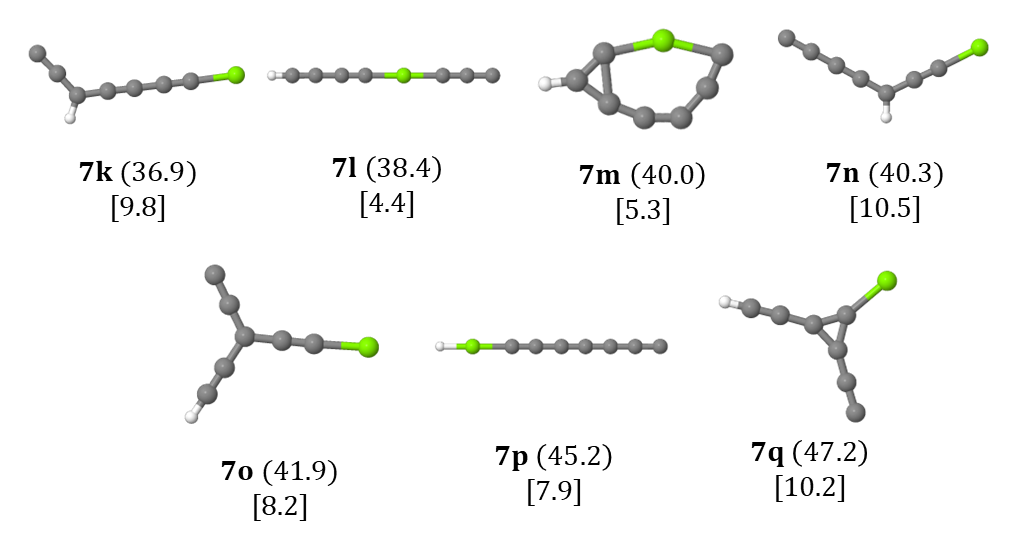


Figure S7 Additional low-energy isomers for MgC_7_H obtained at the UB3LYP/aug-cc-pVTZ level. Energies in kcal/mol (in parentheses) obtained at the CCSD(T)/aug-cc-pVTZ level with ZPE correction at the UB3LYP/aug-cc-pVTZ level. Dipole moments (in square brackets) also obtained at the CCSD(T)/aug-cc-pVTZ//UB3LYP/aug-cc-pVTZ level.


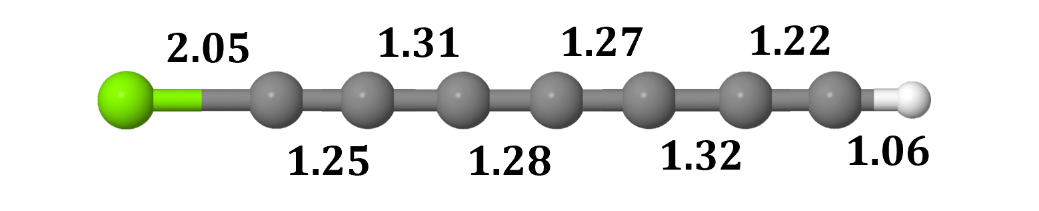


Figure S8 Structural parameters obtained at the UB3LYP/aug-cc-pVTZ level for structure 7lin. Bond lengths (Å) are reported in black.

**Table S3** Enthalpies at 0 K for different dissociation reactions involving the lowest energy isomers of chemical formula MgC*n*H (*n* =4–7) and their respective anions. Geometries and corrections to enthalpy obtained at the UB3LYP/aug-cc-pVTZ level and energy calculated at the CCSD(T)/aug-cc-pVTZ level for *n* = 4,5 and CCSD(T)/aug-cc-pVDZ for *n* = 6,7. (a) Values reported by Bâldea 2020 (*Mon. Not. R. Astron. Soc.* **2020**, *498*, 4316–4326).

|  | ***n*a → MgC*_n_* + H** | ***n*a → MgC*_n_* + Mg^0^** | ***n*a^–^ → MgC*_n_^–^* + Mg^0^** |
| --- | --- | --- | --- |
| **4a** | 126.8^(a)^ | 75.7^(a)^ | 29.0 |
| **5a** | 97.4 | 119.6 | 48.6 |
| **6a** | 145.8 | 71.2 | 23.0 |
| **7a** | 93.7 | 110.4 | 46.4 |

**Cartesian coordinates**

All cartesian coordinates are given in Å. Level of theory: UB3LYP/aug-cc-pVTZ.

**4a**

Mg 3.787407682 0.000000091 0.000000108

C -0.852429659 -0.000000036 -0.000000156

C 1.734674319 -0.000000050 0.000000044

C -2.059489805 -0.000000033 -0.000000097

C 0.510390532 -0.000000050 -0.000000103

H -3.120553068 0.000000078 0.000000203

**4b**

Mg 0.892552680 0.000354890 -0.000128051

C -2.329055191 -0.000229263 -0.000037964

C 2.972218513 0.629311975 0.000087043

C -1.115637176 0.000397861 -0.000247755

C 2.972763384 -0.629146352 0.000086455

H -3.392842209 -0.000689111 0.000240272

**4c**

Mg -2.454157795 2.987433196 0.000151775

C -4.054314594 1.655716943 -0.000098703

C -0.841944945 1.628560270 -0.000183931

C -3.066283423 0.902065588 -0.000003567

C -1.654118509 0.556496419 0.000081292

H -1.313550735 -0.477652416 0.000053134

**4a - Cation**

Mg 3.677234919 -0.000000218 -0.000000031

C -0.827038138 0.000000290 -0.000000031

C 1.750414340 -0.000000045 0.000000051

C -2.032373474 -0.000000037 -0.000000061

C 0.528551420 0.000000241 0.000000022

H -3.096789068 -0.000000230 0.000000050

**4b - Cation**

Mg 0.953264185 0.000231087 -0.000232835

C -2.393987679 -0.000255778 -0.000040989

C 3.027107339 0.637029778 0.000138426

C -1.147416220 0.000004658 -0.000283912

C 3.027414564 -0.636589394 0.000138936

H -3.466382189 -0.000420351 0.000280374

**4a - Anion**

Mg 3.962529832 -0.000000300 -0.000000375

C -0.887037374 0.000000128 0.000000303

C 1.708325637 0.000000087 0.000000023

C -2.099791222 -0.000000046 -0.000000044

C 0.475131735 0.000000307 0.000000395

H -3.159158607 -0.000000176 -0.000000302

**4b - Anion**

Mg 0.967961459 0.000328711 -0.000098119

C -2.336501796 -0.000252096 -0.000014702

C 2.943284507 0.631474679 0.000069253

C -1.118559212 0.000222680 -0.000224683

C 2.943504181 -0.631184967 0.000068617

H -3.399689139 -0.000589007 0.000199633

**5a**

Mg 1.808174350 0.001315862 0.000101326

C 0.847961964 1.825742686 0.000052004

C -1.015050247 -0.000731468 -0.000053607

C -0.199317848 1.147522221 -0.000014145

C 0.850583222 -1.824522721 0.000023272

C -0.197674361 -1.147815315 0.000035300

H -2.094677079 -0.001511265 -0.000144150

**5b**

Mg 0.541810834 1.259087001 0.000054231

C -0.800704410 -0.647525385 -0.000017224

C 2.428020579 0.206416042 -0.000036739

C 0.320553964 -1.282875817 -0.000066106

C -1.494582662 0.456314339 0.000048367

C 1.543408536 -0.704886169 -0.000060518

H -2.538506842 0.713469988 0.000077989

**5c**

Mg 4.025412886 0.078156039 0.000739119

C -1.819366199 1.047989812 0.000892721

C -0.639085616 0.226951421 -0.000846075

C 1.963125199 0.099041619 -0.000884277

C 0.740418612 0.150593162 -0.002847429

C -1.845311750 -0.346758425 0.001033606

H -2.425193133 -1.255973629 0.001912335

**5d**

Mg 3.408797449 -0.658275540 -0.000000342

C 1.571477464 0.386710652 -0.000001589

C 0.221815959 0.492974304 0.000000259

C 0.971731539 1.685120849 0.000000499

C -1.017534674 -0.128151366 0.000001151

C -2.099674446 -0.659327279 0.000000124

H -3.056613290 -1.119051619 -0.000000103

**5e**

Mg 2.019677213 1.042748475 0.000539086

C 2.065219986 -1.264351487 0.000122724

C -1.530253480 0.260804265 -0.000233855

C 0.834210585 -1.039333037 -0.000023869

C -0.298063376 -0.269020294 -0.000180074

C -0.492952679 1.164297202 -0.000040143

H -2.597838249 0.104854875 -0.000183869

**5f**

Mg 0.343970877 0.004527190 0.000520614

C 2.346227704 0.764693788 -0.000014031

C -1.663976926 0.003615955 0.000838655

C 3.449733958 -0.002050488 -0.000313101

C 2.342758153 -0.763659525 0.000011208

C -2.877391658 -0.001670417 -0.000151249

H -3.941322107 -0.005456503 -0.000892097

**5g**

Mg -0.064791157 0.006109668 0.000475646

C 3.232929102 0.000757557 -0.000271634

C -2.066999618 0.000290627 -0.000005895

C 1.970278982 0.003660010 -0.000907312

C -3.280236420 -0.002190278 0.000175755

C 4.553201963 -0.003807295 0.001216306

H -4.344382852 -0.004820288 -0.000682867

**5h**

Mg 3.830607708 0.478819164 0.000374309

C 1.810523380 0.004335470 -0.001099092

C 0.602944832 -0.204283537 0.001124888

C -1.741036555 0.445040655 0.000504198

C -2.690799532 1.289888787 -0.000084540

C -0.767527927 -0.479450306 0.000151529

H -1.044711906 -1.534350234 -0.000971291

**5i**

Mg 2.84318623245893 1.01580109393240 0.00000117379421

C 1.04135995781578 -0.12493595210794 -0.00003189566020

C -1.44463174972080 0.86496136324358 0.00013709026829

C -2.40965564507004 1.67132607182887 -0.00009543063075

C -0.38777505671585 0.04217376387102 0.00005037452614

C 0.22169069886693 -1.19699868369006 -0.00001504422559

H 0.13582556226506 -2.27232765697787 -0.00004626807210

**5j**

Mg -3.043393040 -0.000000089 0.000000012

C -0.998363580 -0.000000340 -0.000000446

C 4.129110782 0.000000213 0.000000634

C 2.830693340 0.000000141 0.000000061

C 1.559521892 -0.000000060 -0.000000379

C 0.246297430 -0.000000324 -0.000000571

H -4.723866824 0.000000459 0.000000689

**5lin**

Mg 4.460756353 0.000001143 0.000000096

C 1.151425989 -0.000000730 0.000000012

C -1.451467856 -0.000000736 -0.000000292

C 2.414738367 0.000000166 0.000000109

C -2.685584755 0.000000030 -0.000000157

C -0.143261005 -0.000001109 -0.000000142

H -3.746607093 0.000001237 0.000000374

**5a - Cation**

Mg 1.97046840063048 0.00141482376891 0.00006099682298

C 0.82451282914806 1.78992903921430 0.00008103702730

C -1.03020672776173 -0.00074554511486 -0.00009191267726

C -0.23833879444113 1.13495078336250 0.00000112825035

C 0.82710242205936 -1.78873318157410 0.00008332068206

C -0.23670190770894 -1.13530095834150 0.00000030930832

H -2.11683622192611 -0.00151496141524 -0.00013487941377

**5b - Cation**

Mg 0.58513415891677 1.40681005199606 0.00005692648904

C -0.79220718297876 -0.64715236737203 -0.00001768928233

C 2.40348646890363 0.18338479529148 -0.00003368682484

C 0.32489438368153 -1.33305574309239 -0.00007001673178

C -1.49198456187980 0.41474019259684 0.00004631653252

C 1.50307703826611 -0.72484597557630 -0.00006139707011

H -2.53240030490949 0.70011904615635 0.00007954698751

**5c - Cation**

Mg 3.92121241285483 -0.15437073427145 -0.00085883899868

C -1.83164425171693 1.01649579689844 -0.00020450469914

C -0.62675000259042 0.27210319169316 -0.00025182637438

C 1.96715563421581 0.30231662876545 -0.00019245586844

C 0.74524648036040 0.22318186037178 -0.00100298261970

C -1.83040690778600 -0.35589617879818 0.00079910754057

H -2.34481336533771 -1.30383056465919 0.00171150101978

**5d - Cation**

Mg 3.42240179650209 -0.56690655019780 -0.00000020285399

C 1.57332337400026 0.37625200390760 -0.00000178091539

C 0.22517036575541 0.43051539158975 0.00000010361305

C 0.99789358739419 1.63519397368675 0.00000060603336

C -1.01346619388867 -0.15461729507185 0.00000113623684

C -2.11455791166995 -0.64646135150105 0.00000026864781

H -3.09076501799333 -1.07397617231339 -0.00000013086169

**5e - Cation**

Mg 1.77965083026707 0.97605912547090 0.00038767605190

C 2.11077575578994 -1.12199359672516 0.00013760844191

C -1.49363062733215 0.29366921172216 -0.00016402658304

C 0.86067445099496 -1.08599695512556 -0.00003617178996

C -0.30711721748072 -0.36341811508770 -0.00010909553145

C -0.37832473858910 1.07251122804847 0.00009198915758

H -2.57202845364999 0.22916910189687 -0.00030797984695

**5f - Cation**

Mg -0.14696275138153 0.00037230451387 0.00104368419737

C 4.60661043224110 0.33038447035161 -0.07827018518112

C -2.10274766765309 -0.14311724631097 0.02895657771487

C 3.28932308921637 0.23074588382603 -0.05030200570956

C 2.04122201581528 0.14621684201916 -0.03118154192301

C -3.31242509012399 -0.23987105487475 0.05439891470293

H -4.37502002821414 -0.32473119952495 0.07535455619853

**5g - Cation**

Mg -0.14649397738356 0.00584153824514 0.00074107366052

C 3.29794592784614 0.00066217314095 -0.00017263747251

C -2.10790271386997 0.00054062306557 -0.00008750748988

C 2.04673197486228 0.00326717498122 -0.00033224316148

C -3.32167049433062 -0.00214101914973 -0.00023768722880

C 4.61921616126651 -0.00344310745156 0.00072239784890

H -4.38782687829078 -0.00472738283157 -0.00063339605675

**5h - Cation**

Mg 3.64636847336509 0.61669819414079 0.00089782233580

C 1.81655348729657 -0.04670728510133 -0.00043838977061

C 0.61668982401842 -0.26335085147333 -0.00086639571894

C -1.67752322896098 0.43998592962279 0.00026300785559

C -2.56409804120667 1.36941698326377 0.00031005036275

C -0.77042054583195 -0.53398644822000 -0.00018439434898

H -1.06756996878047 -1.58205652233267 0.00001829928439

**5i - Cation**

Mg 2.88050242488134 0.94426209291492 -0.00002214201267

C 1.02837418716412 -0.07921700888515 0.00000533070680

C -1.48526000712569 0.81203705204749 0.00015108845186

C -2.42885503924755 1.67660803001849 -0.00011545449044

C -0.39851896245444 0.06294840626840 0.00006169055275

C 0.26303730741899 -1.17229843475728 -0.00001921868471

H 0.14072008926324 -2.24434013750688 -0.00006129452358

**5j - Cation**

Mg -3.13373085592112 -0.00000016534181 0.00000000560211

C -0.95721534652532 -0.00000018857243 -0.00000068604820

C 4.15741992469784 -0.00000018830386 0.00000078974436

C 2.84868755138706 0.00000019817978 0.00000012247501

C 1.59266008291705 0.00000020685053 -0.00000042122231

C 0.28472878601897 -0.00000005185828 -0.00000077968915

H -4.79255014227448 0.00000018904607 0.00000096913817

**5a - Anion**

Mg 1.74377186106310 0.00127986349852 0.47777606254521

C 0.84541385166568 1.86689194293030 -0.19627811486392

C -0.99942109558604 -0.00070709550565 -0.10325888171676

C -0.21865322449866 1.16277492546065 -0.14503377747721

C 0.84809457016508 -1.86570066597233 -0.19623730129338

C -0.21697716892329 -1.16309074016656 -0.14509981188597

H -2.00222879388589 -0.00144823034493 0.30813182469202

**5b - Anion**

Mg 0.50764850221410 1.21798743201457 0.00122989929058

C -0.79295760987479 -0.66189066239787 0.00012380580338

C 2.43191988276773 0.22293139117379 -0.00091695858409

C 0.32451211939432 -1.28837177561188 0.00054543058522

C -1.49264584372650 0.46763878245751 0.00002289454748

C 1.55208144179739 -0.69968755054541 -0.00034777521383

H -2.53055849257226 0.74139238290928 -0.00065729632874

**5c - Anion**

Mg 4.21672303408346 0.03075122564749 0.00021396996027

C -1.88207156403345 1.03613479060782 0.00052042758118

C -0.66910736804180 0.25102649546950 -0.00071255144687

C 1.93696688713094 0.11514655705748 -0.00059654090652

C 0.70467551965866 0.18867264439059 -0.00228923423758

C -1.87022219664590 -0.35125840887647 0.00094053197160

H -2.43696431215193 -1.27047330429641 0.00192339707791

**5d - Anion**

Mg 3.43808094644981 -0.66425360995027 -0.00000035193105

C 1.57537694159467 0.38813102128811 -0.00000156834891

C 0.21753103669179 0.49484048829885 0.00000024355386

C 0.96183247485911 1.68513131973926 0.00000049879414

C -1.02216968332607 -0.12694450945907 0.00000115011154

C -2.10767348389359 -0.65889289699069 0.00000012630344

H -3.06297823227572 -1.11801181282618 -0.00000009858303

**5e - Anion**

Mg 4.11007240693060 -0.56197626166203 0.24364398689209

C 1.94898860937781 -0.04767524480382 -0.22615699759300

C -1.88007876124295 -0.13351605912505 0.02892702577627

C 0.73255955134877 0.14414346170965 -0.12009770661569

C -0.62379415665451 0.34336323005365 -0.03024771563180

C -1.74991752761734 1.24575926510297 0.07052985542534

H -2.53783012214236 -0.99009839107538 0.03340155164678

**5f - Anion**

Mg 0.39112887406946 0.00173634901529 0.00040962969608

C 2.30886717430216 0.72616637097238 -0.00001383854520

C -1.67986338255891 0.00127421022552 0.00075502504818

C 3.52908079336103 -0.00004397048595 -0.00022694860677

C 2.30813164269182 -0.72506204625100 0.00001711212152

C -2.89705676344100 -0.00120287823925 -0.00014428033435

H -3.96028833852455 -0.00286803523699 -0.00079669937945

**5g - Anion**

Mg -0.01610437528957 0.00446176877382 0.00069226932970

C 3.24726083102013 0.00066681578038 0.00002475520877

C -2.07581122356441 0.00009214294709 -0.00020705862769

C 1.94875258966960 0.00263797855417 -0.00092063809619

C -3.29340014736072 -0.00183193761798 -0.00016488945343

C 4.54593166901881 -0.00218718498707 0.00099698686426

H -4.35662934339384 -0.00383958345040 -0.00042142512542

**5h - Anion**

Mg 3.66974772521349 0.40092688189495 -0.46973309847915

C 1.83646642150196 -0.00684155424913 0.49395243683343

C 0.63513411367326 -0.17366653869455 0.21569152258461

C -1.73759873154834 0.46868393534802 -0.02417309574583

C -2.73593085005674 1.23564023607813 -0.06580795279465

C -0.70853597245062 -0.43690183813849 0.00296099527030

H -0.95928270643299 -1.48784112233894 -0.15289080766871

**5i - Anion**

Mg 3.30040087268400 0.70591572469226 -0.00000804782526

C 0.99709200077227 0.06528539485348 0.00000249125445

C -1.60138143402081 0.83618181437682 0.00009706715184

C -2.67658580554081 1.47585191051742 -0.00007112870722

C -0.44487201375969 0.12364333590694 0.00004281073589

C 0.22584137658935 -1.06367737360706 -0.00001430978324

H 0.19950500317571 -2.14320080663986 -0.00004888282646

**5j - Anion**

Mg -3.00388738721859 -0.00000008184444 -0.00000021490617

C -1.02771958625013 -0.00000035410046 -0.00000061440537

C 4.12200848894008 0.00000046678265 0.00000096097383

C 2.84345702532214 0.00000011890722 -0.00000010128702

C 1.53880750583789 -0.00000027765808 -0.00000055374646

C 0.24526392915249 -0.00000049640594 -0.00000053001610

H -4.71792997548389 0.00000062431903 0.00000105338728

**6a**

Mg 5.116151064 -0.000002032 -0.000004100

C -2.091383607 0.000000844 0.000000963

C 0.480266207 0.000001650 0.000003419

C 1.832679316 0.000000719 0.000001716

C -3.299536419 -0.000000776 -0.000001252

C 3.059972941 -0.000000498 -0.000000763

C -0.737312997 0.000002037 0.000003451

H -4.360836507 -0.000001944 -0.000003434

**6a - Anion**

Mg 5.327642818 -0.000002660 -0.000004150

C -2.122212502 0.000000789 0.000001129

C 0.453446792 0.000002227 0.000003181

C 1.801938467 0.000001127 0.000001716

C -3.335177804 -0.000001215 -0.000001141

C 3.039487900 -0.000000536 -0.000000696

C -0.770272766 0.000002202 0.000003404

H -4.394852904 -0.000001935 -0.000003442

**6b**

Mg 1.839134494 0.732582870 -0.000013362

C -1.320630622 -0.037462181 0.000030969

C 1.986676286 -1.322990659 0.000003369

C 0.310292992 2.056283899 -0.000119071

C -0.594604727 -1.164471363 0.000013388

C -0.552885968 1.166794271 0.000086983

C 0.734876112 -1.403378354 0.000019129

H -2.402858566 -0.027358484 -0.000021405

**6c**

Mg 0.249479656 1.389876782 0.000008712

C 1.208049350 -1.441133538 0.000195104

C -1.712019832 0.547720617 -0.000263917

C 2.272886075 0.952380108 0.000253887

C -0.086195760 -1.409207033 0.000066794

C 1.925513037 -0.249823978 0.000257804

C -1.096206664 -0.590632838 -0.000109380

H -2.761505863 0.800819882 -0.000409005

**6d**

Mg 2.294049677 0.007275370 0.002498519

C 0.278605779 0.010515154 0.004389784

C -3.517224464 -0.004170759 -0.001504362

C -2.309186382 0.001279437 0.000227537

C 4.385179637 -0.637152535 -0.001957362

C -0.945652168 0.006775942 0.002614872

C 4.392434935 0.624966712 -0.002194109

H -4.578207014 -0.009489320 -0.004074880

**6e**

Mg -0.787503557 0.000000795 -0.000001304

C 1.245214074 0.000000966 -0.000001475

C 2.490062073 0.000000485 -0.000000779

C 5.094848915 -0.000001019 0.000001774

C 3.814095343 -0.000000260 0.000000336

C -2.788700981 0.000000179 -0.000000560

C -4.001916762 -0.000000341 0.000000397

H -5.066099106 -0.000000805 0.000001612

**6f**

Mg 3.155762292 1.228206965 0.000145696

C -0.376125333 -0.133518874 0.000637441

C 2.137101492 -0.663504166 -0.000662881

C -4.060819203 0.899761249 -0.000232973

C -1.587348783 0.192712061 0.000508196

C -2.842266140 0.543937450 0.000157193

C 0.874346368 -0.482258461 0.000919604

H 2.699349307 -1.585336225 -0.001472275

**7a**

Mg 1.621487725 1.240701248 -0.000054403

C 2.788664857 -0.473719465 -0.000064065

C -0.225508255 2.082092126 -0.000165099

C -1.042150269 1.145422694 0.000327814

C -1.714086341 -0.090023368 0.000087101

C -0.773683855 -1.105791571 0.000051277

C 1.723226990 -1.130458136 -0.000102861

C 0.408476680 -1.459287337 -0.000026630

H -2.786427531 -0.208936192 -0.000053133

**7a - Anion**

Mg 1.362840067 0.997158046 0.000285084

C 2.909172147 -0.509662400 0.000962326

C -0.269552983 2.202650715 -0.000630864

C -1.064815092 1.215805503 -0.000347269

C -1.716259516 -0.056594823 -0.000107349

C -0.721133330 -1.029750026 -0.000601861

C 1.821977294 -1.171353752 0.000002655

C 0.468268903 -1.431690789 -0.001209847

H -2.790497491 -0.216562474 0.001647125

**7b**

Mg 2.783212011 -0.000066146 0.000061694

C -0.074437423 0.000004926 0.000402606

C 1.818302963 1.814326622 0.000182184

C 0.767900921 1.149704824 0.000344271

C 0.767852671 -1.149729360 0.000352951

C -2.676138100 0.000055220 -0.000951249

C -1.467024279 0.000032236 0.000709148

C 1.818214221 -1.814415790 0.000178769

H -3.737882985 0.000087468 -0.001280373

**7c**

Mg 0.128808306 1.518735731 -0.000662983

C -0.697052348 -1.564600385 0.000008180

C 2.164318213 1.384627565 0.000327031

C -1.421942533 -0.491779237 -0.000741461

C -1.860430790 0.735829068 -0.001050980

C 2.126207418 0.133577312 0.001033913

C 1.817124804 -1.211623392 0.001359519

C 0.619336605 -1.604892557 0.000984802

H -2.876369675 1.100125896 -0.001258023

**7d**

Mg 1.324074480 1.204446119 -0.001566976

C -1.875705760 0.110061694 0.000413194

C -0.517239810 -0.038652051 0.000897092

C -3.070611743 0.321859841 -0.002389294

C 2.750656829 -0.461662567 0.001287640

C 3.409241871 0.620182851 -0.000913975

C 0.433063423 -0.961275150 0.002763766

C 1.666380023 -1.276692007 0.002979190

H -4.119859311 0.481731270 -0.003470637

**7e**

Mg 1.700241 1.502919 -0.048254

C 2.606987 -0.321481 0.010534

C -0.215351 2.203381 0.004565

C -0.768370 1.066405 -0.005508

C -1.268075 -0.160009 0.018551

C -0.986054 -1.490547 -0.008098

C 1.541187 -1.006967 0.030675

C 0.382756 -1.641740 0.050333

H -1.706760 -2.294731 -0.024517

**7f**

Mg 4.845901172 -0.505277155 -0.000648439

C 2.865545401 0.076663206 0.000407753

C -2.808142105 -1.187055931 0.000068107

C -0.927401166 0.595534171 -0.000846361

C 0.388311458 0.885119661 -0.000220603

C -1.933380403 -0.360230866 -0.001222311

C 1.700575219 0.451350976 0.000274369

C -0.548204477 1.957149201 -0.000619941

H -3.583205101 -1.913253263 0.002807426

**7g**

Mg 2.288994112 -0.000478156 0.054194668

C -1.987071737 0.000423466 -0.047935164

C 1.549267870 1.919600754 -0.058272701

C 0.374341048 1.506963342 -0.120006282

C 0.373648022 -1.507141171 -0.120363740

C -0.725680504 -0.679590736 -0.212710241

C -0.725354507 0.679936987 -0.212499671

C 1.548442772 -1.920167811 -0.058834941

H -2.696587075 0.000453325 0.776428071

**7h**

Mg 4.355961962 1.131224567 0.000426373

C 2.808309133 -0.310690344 0.000735954

C -3.483968200 0.637867841 0.000147825

C -2.315047857 0.333936489 -0.000141842

C -1.004254927 -0.006645486 -0.000520945

C 0.172973040 -0.315506726 -0.001038585

C 2.480893341 -1.694225449 0.000181598

C 1.497977712 -0.675614827 -0.000322587

H -4.512844204 0.899653934 0.000532209

**7i**

Mg -1.491168137 0.025460800 -0.000773236

C 3.097947855 0.003503746 -0.000207374

C 0.540561957 0.015430241 0.000945337

C 5.667528976 -0.005568372 -0.001635067

C -3.492527794 0.003732935 -0.001506742

C 1.784306365 0.010451715 0.001329535

C 4.368562489 -0.001559300 -0.000587762

C -4.705594390 -0.016755003 0.000119674

H -5.769617321 -0.034696763 0.002315635

**7j**

Mg 1.711012730 0.003573287 -0.002943168

C -4.089332166 -0.000034740 0.002794697

C 4.813367704 -0.001586018 0.003184837

C 3.707976649 -0.765569582 0.000977573

C 3.708545165 0.762787385 0.001015488

C -1.520179166 0.000017263 -0.003137883

C -2.882861366 -0.000167347 -0.000220139

C -0.297865734 0.000791363 -0.006396597

H -5.150663816 0.000188389 0.004725192

**7k**

Mg 5.292685975 0.430077003 -0.000065654

C 0.681236855 -0.161145177 -0.000774748

C -2.824300362 0.496759325 0.000373726

C -0.531111763 -0.293186092 0.000007616

C -1.907375930 -0.486628544 0.001059845

C 3.238964746 0.146932753 -0.001331578

C -3.726275008 1.392511173 -0.000462845

C 2.020058002 -0.000990295 -0.001379318

H -2.243882515 -1.524330145 0.002572956

**7l**

Mg 1.390934311 -0.000000935 0.000000797

C -0.611212123 0.000001742 0.000001271

C -1.833639228 0.000002089 0.000000738

C -4.402034271 -0.000000761 -0.000000700

C 6.006254336 -0.000000025 -0.000000934

C 4.685794716 -0.000000204 -0.000000543

C -3.195837882 0.000000997 0.000000200

C 3.423351362 -0.000000701 0.000000047

H -5.463611222 -0.000002202 -0.000000877

**7m**

Mg 1.026266574 1.304647911 -0.000081029

C -1.135101822 -0.586356067 -0.001189205

C -2.126372891 0.372543810 -0.000969594

C -1.054163779 1.154778516 -0.000048656

C -0.001651803 -1.233510178 0.000150615

C 2.958508587 0.533014453 0.000308943

C 2.291736663 -0.534431675 0.000515133

C 1.247509802 -1.418309576 0.002281735

H -3.206731329 0.407622806 -0.000967941

**7n**

Mg 4.616348189 1.064361402 0.000992931

C -3.019437755 0.874646762 -0.000673009

C -0.772318330 -0.342695244 -0.000184243

C 2.762262156 0.132109367 -0.004011305

C -1.873538897 0.251291425 -0.000387924

C 1.649143818 -0.380259122 -0.002195352

C 0.393707250 -0.997121631 0.001196670

C -4.136979908 1.484730894 0.000014818

H 0.380813476 -2.087063853 0.005247414

**7o**

Mg 4.389209994 0.007919929 -0.006138111

C -0.896157393 1.629964280 -0.000563046

C 1.101579733 0.263899738 0.010086094

C -0.301841024 0.412139753 0.002966029

C -1.449817455 2.771227518 -0.006107567

C -1.083307986 -0.776622764 -0.001232258

C 2.318420523 0.137221483 0.013308701

C -1.741888127 -1.782578971 -0.004672351

H -2.336198266 -2.663170967 -0.007647492

**7p**

Mg -4.378710559 0.000000145 -0.000000087

C 4.050164722 0.000000276 -0.000000346

C -2.334479408 -0.000000544 -0.000000251

C 2.770799249 -0.000000005 0.000000077

C 0.228862292 -0.000000683 0.000000646

C 1.478128490 -0.000000305 0.000000509

C -1.096665294 -0.000000153 0.000000478

C 5.341329478 0.000000469 -0.000000502

H -6.059428970 0.000000799 -0.000000525

**7q**

Mg 2.850844126 2.051660977 0.000126346

C 1.229179140 -1.991670838 0.000469526

C -2.601186428 0.963161425 -0.001117623

C 0.869948269 -0.702397173 0.000811815

C 1.595657518 -3.195315470 -0.000869570

C -0.094821768 0.308932704 0.000768304

C -1.431780438 0.678880676 0.000394454

C 1.217237121 0.678278137 0.001565410

H -3.635077540 1.208469562 -0.002148662

**7lin**

Mg 5.773518321 0.001539596 0.002349694

C 3.721479904 -0.000871006 -0.002245340

C -2.705323047 -0.000567063 0.000471918

C 2.471674423 0.000350244 -0.000976220

C 1.158656050 0.001660268 -0.000861545

C -3.929722288 -0.001878391 0.000618656

C -0.116792538 0.001647945 -0.000493377

C -1.382540146 0.001095601 0.000661903

H -4.990950679 -0.002977194 0.000474311
